# Carcinogenesis and chemo-prevention

**DOI:** 10.1038/sj.bjc.6690733

**Published:** 1999-10

**Authors:** British Association for Cancer Research, Royal Society of Medicine (Oncology Section)

**Affiliations:** Joint Special Conference 7–8 December 1998, Commonwealth Institute, Kensington High Street, London, W8, UK


*British Journal of Cancer* (1999) **81**(4), 565–585
© 1999 Cancer Research Campaign
Article no. bjoc.1999.0733

Abstracts of invited papers

### Carcinogenesis and chemo-prevention

British Association for Cancer Research and Royal Society of Medicine (Oncology Section): Joint Special Conference, 7–8 December 1998, Commonwealth Institute, Kensington High Street, London W8, UK

### S1 Mechanisms of chemical carcinogens: role in human risk assessment

*LL Smith*
Zeneca Central Toxicology Laboratory, Zeneca, Alderley Park, Macclesfield, SK10 4TJ, UK

The extrapolation to man of data generated in experimental animals is the quintessence of toxicology. It is now generally accepted that for many drugs, industrial chemicals or pesticides, the most scientifically rational approach to establishing the likely risk of cancer to man depends on an understanding of mechanism of toxicity in experimental animals, combined with an appreciation of the relevant human biochemical and physiological pathways.

The drug, tamoxifen, is a genotoxic rat carcinogen which has a favourable risk benefit profile in the treatment of breast cancer patients. In order to establish whether the ability of this drug to provoke liver cancer in rats is relevant to human population, a through investigation of its mechanism of carcinogenicity in rats has been undertaken. This analysis is of particular relevance for the utility of this drug for medical indications other than the treatment of breast cancer, and these studies, taken with many years of clinical use of the drug, enable a comprehensive risk assessment to be established.

The industrial solvent, methylene chloride, causes tumours in mouse lung by a mechanism which is arguably qualitatively different to humans. Certainly there are major quantitative differences in the metabolism of methylene between man and animals, which greatly alter the likelihood that this solvent will present the same hazard to different species.

A number of drugs, industrial chemicals and pesticides cause peroxisome proliferation in the liver of rodents. This phenomenon has long been linked with the formation of the liver tumours and as the understanding of the mechanism of peroxisome proliferation has developed, so has our appreciation of the differences between human liver and the liver in those species which develop peroxisomes. It now appears highly unlikely that chemicals which produce liver tumours solely through the formation of peroxisome proliferation are likely to pose a risk of humans and this has considerably altered the risk assessment paradigm for this class of chemical.

These examples reinforce the assertion that the most meaningful risk assessments for human toxicity depend on a comprehensive understanding of the biochemical basis of mechanism of carcinogenicity.

### S2 Risk factors in alkylating agent carcinogenesis: ménage à trois?

*GP Margison[1], AC Povey[1,2] and PJ O'Connor[1]*
[1]CRC Section of Genome Damage and Repair, Paterson Institute for Cancer Research, Christie Hospital, Manchester M20 4BX, UK; [2]School of Epidemiology and Health Science, Medical School, University of Manchester, Manchester M13 9PT, UK

Exposure, uptake, distribution and metabolic or spontaneous activation of carcinogens are clearly required to generate the DNA lesions that can give rise to malignant transformation. DNA replication on an appropriately damaged template is obligatory for instituting mutations, and DNA repair processes can prevent this, but generally only if they operate before DNA replication. Recent studies using alkylating agents in model systems reaffirm the concept that whilst the detection and quantitation of specific DNA lesions is one important aspect of risk assessment, DNA repair and DNA replication must also be taken into consideration as these can be overriding determinants of the frequency with which damaged target cells undergo the events that result in malignant transformation.

Supported by the Cancer Research Campaign and MAFF.

### S3 Regulation of p53 function

*A Phillip[1], S Bates[1], M Kubbutat[1], R Ludwig[1], P Clarke[2], F Stott[2], G Peters[2] and K Vousden[1]*
[1]ABL Basic Research Program, NCI-FCRDC, Frederick, MD 21702, USA; [2]ICRF, 44 Lincoln's Inn Fields, London WC2A 3PX, UK

*p53* is one of the most frequently mutated tumour suppressor genes in human cancers, and loss of p53 function is thought to contribute to the development of most human malignancies. Under normal circumstances p53 is a short-lived protein which is maintained at very low levels. Activation of a p53 response is mediated, at least in part, by stabilization of the protein and accumulation within the cell. We have recently identified the cellular protein Mdm2 as a regulator of p53 stability. Co-expression of Mdm2 with p53 results in extremely rapid degradation of p53 through the proteasome, and this degradative pathway may contribute to the maintenance of low p53 levels in normal cells. Interestingly, *Mdm2* is transcriptionally activated by p53 and may therefore play a role in controlling the extent and duration of the p53 response.

Since p53 protein can be stabilized following DNA damage, it seems likely that mechanisms which allow p53 to become resistant to degradation by Mdm2 exist. Degradation depends on the interaction between p53 and Mdm2, and modifications such as phosphorylation, which have been shown to regulate p53/Mdm2 binding, could also modulate p53 stability. However, there are additional mechanisms for the control of p53 degradation and we have shown that regions of both p53 and Mdm2 distinct from the domains required for the interaction of the two proteins are also required for degradation.

p53 can be activated by many diverse signals and stabilization may occur through several mechanisms. In recent studies we have identified $p14^{ARF}$, which is expressed from the *CDKN2A* locus which also encodes the $p16^{INK4A}$ protein, as a protein which can inhibit Mdm2-targeted degradation of p53 and therefore allow accumulation of the p53 protein. $p14^{ARF}$ binds to a region of Mdm2 distinct from the p53 binding domain and does not directly interfere with the interaction of p53 with Mdm2. $p14^{ARF}$ can be detected in a trimeric complex with Mdm2 and p53, and leads to the stabilization of both p53 and Mdm2. Interestingly, $p14^{ARF}$ does not appear to contribute to the activation of p53 in response to DNA damage, but the observation that deregulated E2F1 expression strongly activates $p14^{ARF}$ expression indicates that $p14^{ARF}$ participates in the activation of p53 in response to abnormal proliferative signals.

This research was sponsored by the NCI, DHHS under contract with ABL.

### S4 Micronutrients prevent cancer and delay aging

*BN Ames*
University of California, Berkeley, CA 94720, USA

Approximately 40 micronutrients are required in the human diet. Deficiency of vitamins folic acid, B12, B6, C, or E, or iron, selenium, or zinc, mimics radiation in damaging DNA by causing single- and double-strand breaks, or oxidative lesions, or both. The percentage of the US population that is deficient for these eight micronutrients ranges from 5% to 20% for each, and may comprise in toto a considerable percentage of the US population. Folate deficiency occurs in approximately 10% of the US population, and in two small studies, nearly half of low income (mainly African-American) elderly and adolescents. Folate deficiency is shown to cause extensive incorporation of uracil into human DNA (4 million/cell), leading to chromosomal breaks [1]. This mechanism is the likely cause of the increased cancer risk and perhaps cognitive defects in humans. Micronutrient deficiency may explain why the quarter of the population that eats the fewest fruits and vegetables (5 portions a day is advised) has about double the cancer rate of the quarter that eats the most.

Aging appears to be in good part due to the oxidants produced as by-products of normal metabolism by mitochondria [2,3]. In old rats mitochondrial membrane potential, cardiolipin levels, respiratory control ratio and overall cellular $O^2$ consumption declines, and the level of oxidants (per unit $O^2$) increases [2]. The effectiveness of the normal mitochondrial metabolites (conditional micronutrients) acetyl carnitine and lipoic acid in restoring these functions (when fed to old rats for a few weeks) to the level of a young rat and apparently rejuvenating the rats [4] suggests a plausible mechanism. Oxidative damage [3] to proteins (the level of oxidized proteins increases with age) and to lipid membranes causes a deformation of structure of key enzymes, with a consequent lessening of affinity ($K_m$) for the enzyme's substrate or coenzyme. Thus increasing levels of the substrate might restore the $V_{max}$ of the enzyme, and thus normal function.

### REFERENCES

(1997) *Proc Natl Acad Sci USA* **94**:3290
(1997) *Proc Natl Acad Sci USA* **94**: 3067
(1998) *Proc Natl Acad Sci USA* **95**: 288
(1998) *Proc Natl Acad Sci USA* **95**: 4562

### S5 Tamoxifen, a carcinogen and chemopreventive agent

*DH Phillips[1], W Davis[1], A Hewer[1], MR Osborne[1], PL Carmichael[2] and HR Glatt[3]*
[1]Institute of Cancer Research, Haddow Laboratories, Cotswold Road, Sutton SM2 5NG, UK; [2]Imperial College School of Medicine, London SW7 2AZ, UK; [3]Deutshes Institut für Ernährungsforschung, D-14558 Potsdam-Rehbrücke, Germany

Tamoxifen is a non-steroidal antioestrogen widely used in the treatment of breast cancer. Preliminary results from a US chemoprevention trial indicate that it prevents breast cancer in some high-risk women, but this effect has not been seen so far in European trials. It is also a potent liver carcinogen in rats and causes endometrial cancer in women. Understanding the mechanisms of these effects is important for assessing the long-term risks of tamoxifen and of its analogues. Tamoxifen has many properties of a genotoxic carcinogen, including formation of covalent DNA adducts in rat liver in vivo and in rat hepatocytes in vitro, but it does not form DNA adducts in human hepatocytes. The metabolite (-hydroxytamoxifen forms the same pattern of adducts as does tamoxifen in rat liver cells, indicating that it is an intermediate in the activation pathway. The major DNA adduct formed in liver is chromatographically indistinguishable from the major product of the reaction of α-acetoxytamoxifen with DNA, (E)-(-(N2-deoxyguanosinyl)tamoxifen. Minor adducts are the *cis*-isomer of this and analogous *cis*-and *trans*-substitution at the N6-position of adenine. Metabolism studies in cell lines expressing different CYP isoforms indicate that α-hydroxylation is carried out primarily by the CYP3A family. In rat hepatocytes, activation of both tamoxifen and α-hydroxytamoxifen to DNA binding products is highly dependent on sulphate, being tenfold higher in medium containing 10 μM sulphate than in sulphate-free medium, and inhibited by 80% by dehydroisoandrosterone-3-sulphate. We expressed rat hydroxysteroid sulphotransferase (hST) a (a liver-specific enzyme) and human sulphotransferases (STs) in bacteria (*S. typhimurium*) and in a mammalian cell line (V79 cells) and tested α-hydroxytamoxifen for DNA adduct formation and mutagenicity, using unmodified cells as controls. In cells expressing rat hST, α-hydroxytamoxifen was mutagenic and formed the same DNA adduct pattern as that found in the liver of tamoxifen-treated rats. α-Hydroxytamoxifen was not activated in cells expressing human STs. This mechanism provides an explanation for interspecies differences in tamoxifen carcinogenicity and suggests that the risk of DNA adduct formation (and cancer) in the human liver is low. It explains why tamoxifen is a powerful carcinogen in rat liver, and why standard short-term tests fail to detect its mutagenicity. Tamoxifen does not form significant levels of adducts in human endometrium or white blood cells.

 © 1999 Cancer Research Campaign

### S6 P53 mutational landscapes: a sense of exposure

P Burns
Molecular Oncology, Department of Pathological Sciences, Algernon Firth Building, University of Leeds, Leeds LS2 9JT, UK

Genotoxic agents leave their stigmata on genetic material in the form of alterations in DNA sequence. The best place to look for these in human tumours is the *p53* gene. A large and invaluable database of > 10 000 *p53* mutations from most types of human malignancy have been collected at IARC. The *p53* mutational landscape found in each tumour-type is the end-product of a number of processes including initial interaction between agent and DNA, repair, polymerase fidelity and biological selection. DNA sequence context has a profound influence on each of these factors. In order to investigate the contribution of putative carcinogens to human carcinogenesis it is advantageous therefore to use the human *p53* gene as a mutational target. In order to fulfil this requirement we have modified Richard Iggo's yeast-based *p53* functional assay for use as a *p53* forward mutation assay. However, problems arise when studying mutational landscapes even when using the most appropriate human target gene as a window. With few exceptions, such as skin cancer and some occupational exposures, the aetiology of cancer is invariably complex. What is the likelihood that we will be able to dissect out the different contributions made to mutational landscapes by multiple agents in a scenario involving complex exposures? This presentation will describe our attempts to understand cancer aetiology by analysing the *p53* mutational spectra generated by a number of well-characterized human carcinogens.

### S7 Inducible expression of antioxidant and detoxication enzymes as a mechanism of cancer chemoprevention

JD Hayes[1], MM Manson[2], GE Neal[2] and A Gallina[3]
[1]Biomedical Research Centre, UK Ninewells Hospital and Medical School, University of Dundee, Dundee DD1 9SY, UK; [2]MRC Toxicology Unit, University of Leicester, Leicester LE1 9HN, UK; [3]Consiglio Nazionale delle Ricerche, Via Abbiategrasso 207, I-27100 Pavia, Italy

There is strong epidemiological evidence that certain forms of cancer can be prevented by dietary intervention, specifically by the high intake of fruit and vegetables. The mechanism whereby diet can protect against carcinogenesis is controversial. It has been proposed that phytochemicals are metabolized within the cell to pro-oxidant species which, in turn, induce the expression of genes that effect adaptive responses to harmful genotoxic agents. Alternatively, it has been suggested that phytochemicals serve to overcome inhibition of apoptosis, thereby allowing the selective removal of damaged and mutated cells. Both induction of detoxication enzymes and modulation of apoptosis are probably important in chemoprevention, and are possibly interdependent.

Our laboratories have investigated the mechanisms of chemoprevention against aflatoxin $B_1$ hepatocarcinogenesis in the rat, and have identified glutathione S-transferase (GST) A5 and aldoketo reductase 7 (AFAR) as highly inducible cytosolic detoxication enzymes that metabolize the mycotoxin. These enzymes have proved to be induced by structurally-diverse chemicals including phenolic antioxidants, isothiocyanates, indoles and lactams. Immunohistochemistry has revealed that the zone of the liver where enzyme induction occurs varies with the agent employed. Moreover, treatment with chemopreventive agent was found to result frequently in nuclear translocation of GST. Immunofluorescence microscopy has shown that AFAR can associate with the Golgi apparatus. Thus, evidence suggests that both GST and AFAR are represented in other subcellular compartments besides the cytoplasm where they are assumed to have additional functions besides that of drug metabolism.

Transcriptional activation of GST genes occurs through the antioxidant responsive element (ARE; 5′-TGACnnnGCA-3′). Work by Yamamoto and his colleagues, using a knockout mouse, has demonstrated that the *N*uclear-factor-erythroid 2-*r*elated *f*actor *2* (Nrf2) mediates GST induction by butylated hydroxyanisole. Studies are underway to determine whether Nrf2 mediates induction by all chemopreventive blocking agents.

### S8 Gene–environment interactions for cancer risk and behaviour

PG Shields
Molecular Epidemiology Section, Laboratory of Human Carcinogenesis, Division of Basic Sciences, National Cancer Institute, Building 37, Room 2C16, Bethesda, MD 20892, USA

Molecular epidemiology methods emphasize *a priori* mechanistic hypotheses and uses biomarkers to explore causative relationships. The goal is to improve risk assessment (both population- and individual-based), elucidate carcinogenic mechanisms and enhance cancer prevention methods. Scientific methods, in general, tend to assume that the population is homogeneous, whether the study relates to a single cloned gene or the study of means in a population. However, the human genome is highly complex and diverse among individuals. One of our fundamental questions is why only one in ten heavy smokers suffer from lung cancer. These methods have allowed us to establish that gene–environment interactions exist, where one's genes can modify the effect of carcinogen exposure. For this presentation, I will focus on our recent studies of breast cancer, tobacco smoking, oxidative damage and diet. I also will discuss recent studies for gene–neurobehavioural risk factors for tobacco addiction.

### S9 The potential for cancer prevention by dietary factors: the role of selenium and flavonoids

PR Harrison
The Beatson Institute for Cancer Research, CRC Beatson Laboratories, Glasgow G61 1BD, UK

It is generally acknowledged that the diet is a significant factor affecting cancer risk and, in particular, that diets rich in fruits and vegetables are associated with a lower risk of many cancers. It is therefore important to identify which dietary factors contribute to this protective effect and elucidate their mechanisms of action, since this might suggest ways to help to protect high-risk groups and lead to the development of synthetic analogues which might be effective chemopreventive agents. This talk will review our group's recent work concerning the potential of flavonoids and selenium compounds as cancer-protective agents.

© 1999 Cancer Research Campaign 

### S10 Mechanisms of action of dietary chemopreventive agents

*MM Manson, A Gescher, EA Hudson and SM Plummer*
MRC Toxicology Unit, University of Leicester, LE1 9HN, UK

Epidemiological studies have long suggested that diet can exert both a negative and a positive effect on health. In particular, diets rich in fresh fruit and vegetables have been shown to be protective against cancer in a variety of target tissues. In addition, beneficial effects of plant and herb extracts have been exploited over the centuries in various forms of medicine. It is now apparent that there are dozens of individual non-nutritive components in food which can exert profound effects on the biochemistry and physiology of cells with which they come in contact. Historically, the effects of chemopreventive agents have been divided into those which block and those which suppress the formation of cancer. Blocking mechanisms prevent carcinogen–DNA interaction by altering activation of the carcinogen or enhancing its conjugation and excretion. They include alterations to phase I (cytochrome P450s) and phase II drug metabolizing enzymes (e.g. glutathione S-transferases, aldehyde reductase, quinone reductase, UDP-glucuronyl transferase, epoxide hydratase) as well as induction of DNA repair capacity. Suppressing mechanisms inhibit promotion and progression of cancer by inhibiting cell proliferation and increasing apoptosis. They can achieve this by modulation of signal transduction pathways and altered transcriptional regulation. Many chemopreventive agents appear to have more than one mechanism of action. The potential role of several such agents, including indole-3-carbinol (from cruciferous vegetables), epigallocatechin gallate (from green tea) and curcumin (from turmeric), in blocking and suppressing carcinogenesis will be discussed.

### S11 Chemoprevention of colon cancer: diet NSAIDs and apoptosis

*C Paraskeva, A Hague, T Crew and DJE Elder*
Department of Pathology and Microbiology, School of Medical Sciences, University of Bristol, University Walk, Bristol BS8 1TD, UK

Colorectal cancer continues to be a major cause of cancer deaths in much of the industrialized world. A number of the genetic changes which occur during colorectal carcinogenesis are thought to uncouple proliferation, differentiation and apoptosis (programmed cell death). The reactivation of apoptosis is thought to be important in the chemoprevention and treatment of colorectal cancer. There is increasing clinical, epidemiological and experimental evidence that dietary and pharmacological intervention may dramatically reduce the incidence and number of colorectal cancer deaths. We have previously reported that butyrate (a fermentation product of dietary fibre/resistant starches) induces apoptosis in colorectal tumour cell lines and that the induction of apoptosis may be part of the chemopreventive action of dietary fibre. Humans who take NSAIDs (non-steroidal anti-inflammatory drugs) on a regular basis have a 40–50% reduction in the relative risk of colorectal cancer. Interestingly, it has also been shown that the traditional NSAIDs and their derivatives (e.g. salicylate, sulindac) induce apoptosis in colorectal tumour cells, suggesting the induction of apoptosis to be a common mechanism for chemopreventive agents. However, the use of traditional NSAIDs – which inhibit both forms of cyclooxygenase (COX-1 and COX-2), a key enzyme in the synthesis of prostaglandins from arachidonic acid – has been restricted because of their gastrointestinal toxicity. A new class of aspirin-like drugs, the so called COX-2 selective NSAIDs which are not as toxic have been developed. These COX-2 selective inhibitors in experimental studies retain much of the chemopreventive activity of the traditional NSAIDs raising the exciting possibility that they could be used to reduce the incidence of bowel cancer in the general population. Recent progress in the mechanism(s) by which dietary factors and NSAIDs are thought to be protective and the possible interactions between diet and pharmacological agents will be reviewed.

### S12 Chemoprevention by diet

*S Bingham*
MRC Dunn Nutrition Unit, Hills Road, Cambridge CB2 2DH, UK

Epidemiological estimates suggest that up to 80% of this breast, bowel and prostate cancer is attributable to diet, which means that it is a potentially preventable disease. However, most of the epidemiological evidence has been derived from prospective studies using simple methods to assess dietary exposure so that estimates of relative risk relate to foods, rather than specific nutrients. High levels of meat, and low levels of vegetables, starch and non-starch polysaccharides (NSP) are associated with higher risk, and there are plausible mechanisms whereby somatic mutations which are relevant to current genetic models of cancer could be initiated or not repaired by factors in these foods, such as endogenous N-nitrosation, heterocyclic amines, ammonia and butyrate formation, and the wide range of potential chemopreventive agents in vegetables. These include phyto-oestrogens which are currently thought to have both oestrogenic and anti-oestrogenic effects at different sites. Whilst various trials of specific food items have been set up, intervention trials of changes in dietary patterns to assess effects on cancer incidence are not at present being conducted.

### S13 Evidence-based chemoprevention: a dream for the future?

*H Vainio*
Institute of Environmental Medicine, Karolinska Institute, Box 210, S-171 77 Stockholm, Sweden

Numerous observational studies conducted during the late 1970s and 1980s found inverse associations between estimated intakes of vitamin A (retinol) and β-carotene and the risk of developing cancer at various sites and other chronic diseases. However, two recent intervention trials indicate that β-carotene supplements potentially increase lung cancer incidence in smokers and in asbestos-exposed workers, and increase mortality from cardiovascular disease. Consequently, many have questioned the value of this whole approach of cancer chemoprevention. Is this an adequate argument, or are we in danger of throwing the baby with the bath water? The reductionism in which the current molecular medicine paradigm has its roots tends to discourage investigators from tackling the complex problems of interfering with tumour development in vivo in humans (preventive trials) or in relevant animal models. It is hard to evaluate how much effect the intense debate about the disappointing results of the β-carotene trials has done to reinforce this, but it is clear that the cost and the difficulty of the human intervention trials have become an increasing

*© 1999 Cancer Research Campaign*

constraint. Yet it is only by conducting randomized intervention trials in humans that the final truth about the biological effects of tentative chemopreventive agents are obtained and the problems of disease biology and systemic toxicity can be adequately addressed. The current fashion in molecular oncology is directed too strongly towards repetitive low-risk artificial in vitro systems. The intervention trials with β-carotene produced data which could not have been obtained otherwise; now it is the challenge for the scientific community to clarify the apparent inconsistency between the trial and observational data. Another challenge the oncology community faces is how to support and encourage new models of chemoprevention in animals and in humans alike, buttressed by an underlying mechanistic understanding of the complex process of carcinogenesis.

© 1999 Cancer Research Campaign

Abstracts of preferred papers

### Carcinogenesis and chemo-prevention

### 1.1 Effects of low level radiation in normal urothelial cells

*CE Mothersill[1], CB Seymour[1], SA Lorrimore[2] and EG Wright[3]*
[1]Radiation Science Centre, Dublin Institute of Technology, Kevin Street, Dublin, Ireland, [2]Radiation and Genome Stability Unit, Medical Research Council, Chilton, Didcot, UK

A major challenge facing radiation protection research is to define the population of cells at risk of surviving with damage. Since there is a large body of evidence that genetic factors are important in determining response to radiation, there is little point in trying to study this in cell lines or inbred mouse strains unless these are selected specifically for their different responses and used only to give pointers to possible mechanisms. Otherwise the genetic variation underlying the variation in human responses, so essential to developing radiation protection actions, will be lost. In an attempt to assess human variation, scores of apoptosis, necrosis and induction of a wide range of proteins were made in primary explant cultures of human normal urothelium and correlated with growth post-exposure to a range of doses of cobalt 60. These data were validated by similar experiments using CBA/H and C57/BL6 mouse strains, known to exhibit genetically determined differences in responses to radiation. The data for human tissues show a wide variation in response with three broad categories being identifiable. The commonest (approx. 60%) had a hypersensitive response involving considerable apoptosis in the low dose region, followed by 'induction' of a survival response at higher doses involving the persistence of abnormal cells and a pattern of gene expression consistent with suppression of apoptosis. The second category (approx. 38%) showed no induction of the survival. The rarest category showed an extremely hypersensitive low dose response followed by induction of a survival response which was also dose-dependent. In the mouse experiments, CBA/H (mice known to be genetically unstable post-irradiation), had lower levels of delayed cell death and apoptosis than C57/BL6 mice (which are more stable). It is concluded that there is a variation in response to radiation between human patient cultures, which is detectable in this system and which is consistent with a pattern of radiation induced delayed death/apoptosis correlating with long-term genomic stability. The mouse experiments demonstrate the importance of genetic factors in determining these responses.

### 1.2 Tamoxifen induces endometrial and vaginal cancer in rats in the absence of endometrial hyperplasia

*P Carthew, LL Smith, RE Edwards and BM Nolan*
MRC Toxicology Unit, Hodgkin Building, University of Leicester, Leicester LE1 9HN, UK

Much controversy has surrounded the finding of an increased risk of endometrial cancer in post-menopausal women taking tamoxifen as an adjuvant therapy for breast cancer (Fornander et al, 1993). The current explanation for this is that tamoxifen acts as an agonist of oestrogen action in the uterus, leading to hyperplasia of the endometrium, and thus promotes endogenous initiated cells to neoplasias, as with hormone replacement therapy (Paganinihill et al, 1969).

We have administered tamoxifen to neonatal rats on days 2–5 after birth, and produced a significant increase in the incidence (26%) of uterine adenocarcinomas, along with a 9% incidence of squamous cell carcinomas of the vagina in the absence of any agonist effect. This demonstrates that an oestrogen agonist effect is not an absolute requirement for the carcinogenic effect of tamoxifen in the reproductive tract of the rat. This does not substantiate the hypothesis that the unopposed oestrogen agonist effect of tamoxifen on the endometrium is responsible for the development of endometrial cancers. The alternatives are that tamoxifen is causing these cancers by its genotoxicity, even though this has been difficult to detect in women or rats (Hemminki et al, 1996; Carmichael et al, 1998); or that modification of 'imprinted' responses, due to tamoxifen treatment, cause reproductive tract cancers to arise at a later time, in the same way as has been proposed for diethylstilboestrol (McLachlan et al, 1998).

### REFERENCES

Carmichael PL, et al. (1998) *Eur J Cancer* **34**: S1, 106
Fornander T, Hellstom AC, Moberger B (1993) *J Natl Cancer Inst* **85**: 1850
Hemminki K, Rajaniemi H, Lindahl B and Moberger B (1996) *Cancer Res* **56**: 4374
McLachlan JA, Newbold RR, Li SF and Negishi M (1998) *APMIS* **106**: 240
Paganinihill A, Ross RK and Henderson BE (1969) *Br J Cancer* **59**: 445

### 1.3 Relative susceptibility of intestinal polyps vs normal mucosa to genotoxic injury from benzo[a]pyrene (BaP)

*A Sattar[1], DH Phillips[2], A Hewer[3] and FC Campbell[1]*
[1]Department of Surgery, University of Newcastle, UK; [2]Institute for Cancer Research, Sutton, UK

Intestinal cancers arise through stepwise genotoxic or mutational damage, in polyps. Polyp capacity for carcinogen metabolism may influence genotoxic injury, but data are lacking. We test the hypothesis that polyps have different metabolic capacity and susceptibility to genotoxic injury, than normal mucosa.

Expression of bioactivation (cytochrome (CYP) p4501A1/1A2) and detoxification enzymes (glutathione S-transferases (GST) α, μ and π class) were assessed in polyps and normal intestinal mucosa from APC$^{min}$ mice, before and 48 h after in vivo exposure to benzo[a]pyrene (100 mg kg$^{-1}$). BaP:DNA adducts were assessed by $^{32}$P post-labelling, in polyps and normal mucosa from APC$^{min}$ intestines.

Eighty polyps and matched normal mucosal specimens were assayed for enzyme expression, while 37 paired samples were assayed for adduct formation. CYP1A1 and 1A2 were undetected

 © 1999 Cancer Research Campaign in untreated polyps or normal mucosa. All GST isoforms were present. BaP induced CYP1A1/1A2 GST α and π to high levels in mucosa but in polyps only CYP1A1/1A2 were induced to low level. BaP:DNA adduct levels were 83.7 ± 15 *polyps* vs 128 ± 19 *mucosa*; $P < 0.0001$).

Intestinal polyps show reduced bioactivation enzyme expression and sustain less DNA damage from BaP, than normal mucosa. Therapy to restrain bioactivation may inhibit carcinogenesis.

### 1.4 The coordinated modulation of the fibroblast growth factor dual receptor mechanism during transformation from human colon adenoma to carcinoma

*GC Jayson[1], C Vives[1], C Paraskeva[2], K Schofield[1], J Coutts[1], A Fleetwood[1] and JT Gallagher[1]*

[1]CRC Dept. Medical Oncology, Christie Hospital and Paterson Inst., Wilmslow Rd, Withington, Manchester M20 4BX, UK; [2]CRC Dept. Colorectal Biology, University of Bristol, Bristol, UK

Basic fibroblast growth factor (bFGF) is the prototypic member of a family of cytokines that express a mandatory dependence on heparan sulphate for their ability to activate the cell surface signal transducing receptor. We have investigated this dual receptor mechanism in a novel model of the transformation from human colon adenoma to carcinoma, in vitro. RT-PCR studies showed that mRNA for FGF receptors 1 and 2 were expressed in both the adenoma and carcinoma cells, whereas immunocytochemistry showed that the expression of the FGF R1 was significantly reduced in the carcinoma cells. The molecular weight of the FGF receptors was compatible with the 3-immunoglobulin loop form of the FGF receptors, suggesting that heparan sulphate was required for their activation by bFGF. We have reported that the composition and sequence of human colon adenoma and carcinoma heparan sulphate (HS) differs in a defined and specific manner (Jayson GC et al (1998) *J Biol Chem* **273**: 51–57). The functional significance of these changes was assessed by affinity co-electrophoresis which showed that the affinity of adenoma HS for bFGF was tenfold greater than that of the carcinoma HS ($K_d$ 220 nM vs 2493 nM, respectively). In addition, Northern blotting studies of the expression of syndecan 1 and 4 mRNA showed that proteoglycan core protein expression was significantly reduced in the carcinoma cells. This reduction in HS-bFGF affinity and proteoglycan mRNA was associated with a reduced biological response to bFGF in the carcinoma cells that could be partially reversed by the addition of exogenous heparin, suggesting that both the proteoglycan and signal transducing receptor control the cells' response to bFGF. The data show that the production of poorly functional HS, lower amounts of proteoglycan and reduced FGF receptor protein accompany transformation in this model. The implication is that either the carcinoma cells are less dependent on exogenous growth factor activity than the adenoma cells or that these alterations contribute to the malignant phenotype. If these findings are common to a range of malignancies the clinical implication is that anti-growth factor strategies should be directed against either preneoplastic lesions or normal cells, such as endothelial cells, that have a greater growth factor requirement.

### 1.5 Insulin-like growth factor binding protein 3 (IGFBP-3) enhances *p53*-dependent apoptosis in oesophageal cancer cells

*AD Hollowood, T Lai, CM Perks, PV Newcomb, D Alderson and JMP Holly*

University Department of Surgery, Bristol Royal Infirmary, Bristol, BS2 8HW, UK

The dysplasia to cancer sequence involves mechanisms in which apoptosis fails to occur appropriately. The tumour suppressor gene *p53* is involved in this sequence in Barrett's oesophagus, being a transcriptional activator of target genes responsible for cell cycle control and apoptosis. IGFBP-3 is one such target gene (Buckbinder et al (1995) *Nature* **377**: 646; Ludwig et al (1996) *Mol Cell Biol* **16**: 4952) with an important link to cancer. Recent studies have shown a strong association between cancer risk and high levels of IGF accompanied by low levels of the main IGF carrier protein IGFBP-3 (Hankinson et al (1998) *Lancet* **351**: 1393; Chan et al (1998) *Science* **56**: 523). Conversely, apoptotic rates are increased experimentally with low levels of IGF-I.

The aim of this investigation was to see if a direct link exists between IGFBP-3, p53 and apoptosis in an oesophageal cancer cell line shown to be devoid of IGF responsiveness.

KYSE 190 oesophageal carcinoma cells were grown in serum-free media for 24 h prior to dosing with IGFBP-3. Cells were subsequently exposed to UV irradiation. Levels of p53 within the cell were detected by Western immunoblotting with a specific monoclonal antibody. Cell death was measured by trypan blue exclusion and apoptosis confirmed by measurement of a pre-G1 peak using flow cytometry.

Levels of p53 peaked within 2 h following exposure to UV irradiation and returned to basal levels after 24 h. Pre-incubation with IGFBP-3 resulted in a 46% increase in the UV induced level of p53 at 2 h with a further 42% increase at 8 h. UV irradiation induced an 86% increase in cell death, with a further 85% increase for cells preincubated with IGFBP-3 ($P = 0.004$).

IGFBP-3, independent of IGF-I, dramatically accentuates UV-induced apoptosis with concomitant increases in p53 concentrations. Delineating the mechanism of action of IGFBP-3 in such a model could lead to a better understanding of the transition from dysplasia to cancer.

### 1.6 p53 protein overexpression correlates with phenol sulphotransferase (SULTIA1) genotype in colorectal cancer

*S Anwar[2], AA Fryer[2], RAHJ Gilissen[1], T Holland[2], DE Bamber[2], JB Elder[2], J Elder[2], J White[2], EJ Bicknell[2], RC Strange[2] and MWH Coughtrie[1]*

Departments of [1]Molecular & Cellular Pathology, University of Dundee, Ninewells Hospital & Medical School, Dundee DD1 9SY, UK; [2]Centre for Cell & Molecular Medicine, School of Postgraduate Medicine, Keele University, North Staffordshire Hospital, Stoke-on-Trent ST4 7QB, UK

Sulphation is a phase II detoxifying reaction, catalysed by sulphotransferases (SULT). Like the other polymorphic, xenobiotic-metabolizing enzymes, these enzymes catalyse the detoxification

© 1999 Cancer Research Campaign as well as activation of various carcinogens. Thus, they are candidates for cancer susceptibility. Mutations in the SULTIA1 gene, which is the major form of phenol sulphotransferase, has been associated with defective enzyme activity and protein stability. p53 protein over-expression is regarded as a marker for mutations in *p53* gene. Accordingly, we examined the p53 over expression in a cohort of patients with mutations in SULT1 AI genotype.

Sixty-eight patients were SULTIA1 genotyped using a PCR assay developed in our laboratory. Wild-type *SULTIA1*1* alleles were differentiated from mutant *SULTIA1*2* alleles using digestion with HaeII. These patients were analysed for p53 protein over-expression using immunohistocytochemistry on resected colorectal tumour samples. Positivity was graded from 0–3.

Our data showed that there was a gene dosage effect for p53 positivity in patients with *SULTIA1*2* alleles. Thus, the propor-tion of patients who were p53-positive was 50% in *SULTIA1*1*/*SULTIA1*1* homozygotes, 64% in *SULTIA1*1*/-*SULTIA1*2* heterozygotes and 100% in patients with *SULTIA1*2*/*SULTIA1*2* homozygous mutant genotypes (trend test: $P = 0.04$).

This data showing, in colorectal tumours, an association between SULTIA1 mutant alleles and over-expression of p53 protein in combination with data showing associations between outcome and expression of p53 suggest that polymorphism at this locus may be a factor determining outcome. Further work is required to elucidate the mechanism of this effect.

### 1.7 Detection of p53 point mutations in circulating tumour cells in patients with colorectal cancer

*SK Jonas, A Tarragona, KA Feldmann, RQ Wharton, K Stupnicka and TG Allen-Mersh*

Department of Gastrointestinal Surgery, Imperial College School of Medicine, Chelsea & Westminster Hospital, 369 Fulham Rd. SW10 9NH, UK

The clinical relevance of identifying disseminated cells from solid tumours in the circulation using RT-PCR and epithelial specific markers has still to be established. Only a very small proportion of these cells will successfully achieve metastasis. To examine the extent to which a subpopulation of these identified cells harbour p53 mutations, we have isolated tumour cells from the circulation of colorectal cancer patients and studied them for their p53 mutational profile.

p53 mutations between exons 4 and 9 were studied in tumour cells isolated from the circulation as well as in matched tumours from 12 colorectal cancer patients. Multiple venous blood samples (three per patient) were collected via a cannula and specifically selected for epithelial cells using immunomagnetic beads labelled with a monoclonal anti-human Ber-EP4 antibody. Total RNA was extracted on these isolated cells followed by two-step RT-PCR for both blood and tissue. All PCR products were initially screened for SSCP by automated gene scanning using a 310 ABI PRISM gene scanner which is based on rapid mutation detection. Products that resulted in mobility shifts were confirmed by automated sequencing.

In five of 12 patients, a total of 13 mutations were detected in the circulation. In three of these patients mutations were detected in known hotspots (codon 248, 273). In a further three patients a total of three mutations were detected. Some of these mutations identified have not previously been reported in colorectal cancer and mutations in blood and matched tissue did not correspond in any patient. No mutations were detected in the blood or tissue from six normal ‘no-cancer’ control patients. Multiple blood sampling increased the frequency of detecting different p53 mutations in circulating tumour cells, suggesting that these cells circulate in clusters constituting distinct cell populations. These clusters may have a selective advantage in achieving metastasis compared with the majority of cells derived from the primary tumour. The difference in mutational pattern between blood and matched tissue from the same patient may be due to sample variation resulting in undetected mutations from different areas of the tumour.

These results demonstrate for the first time that, with the use of immunomagnetic beads for epithelial cell selection, mutations in the p53 gene can be detected in circulating tumour cells. The advantage of convenience in obtaining peripheral blood samples, the greater potential for identifying multiple mutations among cells within the circulation, and difficulties with solid tumour sampling error make circulating tumour cells attractive sources of information about the accumulation of additional mutations during the development of metastasis.

### 1.8 The effect of glutathione S-transferase GSTT1 and GSTM1 genotypes on susceptibility and outcome in colorectal cancer

*J White, S Anwar, M Deakin, A Fryer, J Elder, D Bamber, P Jones, R Strange*

Centre for Cell and Molecular Medicine, School of Postgraduate Medicine, N. Staffordshire Hospital, Stroke on Trent, Staffs, UK.

The genes encoding for the glutathione S-transferase GSTT1 and GSTM1 proteins are polymorphic with common null alleles (Chevenix-Trench et al, 1995; Deakin et al, 1996; Howells et al, 1998). These enzymes are important mediators of in-vivo detoxification since they catalyse the conjugation of glutathione to a wide variety of environmental carcinogens. Consequently, failure to express these enzymes may be associated with impaired carcinogen detoxification and an increased risk of colorectal cancer (Deakin et al, 1996; Cheverix-Trench et al, 1995). Further, recent evidence suggests that these polymorphisms also influence outcome in several common cancers. Accordingly, we have examined the influence of GSTT1 and GSTM1 genotypes on susceptibility and outcome in colorectal cancer.

Blood samples were obtained from cases ($n = 248$) and controls ($n = 510$) and genomic DNA isolated from lymphocytes by phenol/chloroform extraction. Using PCR (Chevenix-Trench et al, 1995; Howells et al, 1998), the null and expressing genotypes for both GSTT1 and GSTM1 were identified in both study and control populations. Clinical data, including follow-up data, was also obtained for the case group.

In the case group, the frequency of GSTT1 null was significantly increased compared to controls ($P = 0.0036$). GSTM1 genotype was not significantly associated with susceptibility or outcome. Analysis of those individuals who underwent ‘curative’ surgery ($n = 144$) showed a median time to recurrence of 3.49 years in those patients who were both GSTT1null/GSTM1null compared to > 10 years for those patients who were non GSTT1null/GSTM1null ($P = 0.037$).

We have confirmed that GSTT1 null is associated with susceptibility to colorectal cancer. We have also shown that patients with both GSTT1 null/GSTM1 null genotypes are at increased risk of recurrence. This data supports our results in ovarian cancer

*© 1999 Cancer Research Campaign*

(Howells et al, 1998) on the importance of interaction between these genes in influencing outcome.

#### REFERENCES

Chevenix-Trench G, Young J, Coggan M and Board P (1995) Glutathione S-transferase M1 and T1 polymorphisms: susceptibility to colon cancer and age of onset. *Carcinogenesis* **16**: 1655

Deakin M, Elder J, Hendrickse C, Peckham D, Baldwin D, Pantin C, Wild N, Leopard P, Bell DA, Jones P, Duncan H, Brannigan K, Alldersea J, Fryer AA and Strange RC (1996) Glutathione S-transferase GSTT1 genotypes and susceptibility to cancer:studies of interactions with GSTM1 in lung, oral, gastric and colorectal cancer. *Carcinogenesis* **17**: 881

Howells EEJ, Redman CWE, Dhar KK, Sarhanis P, Musgrove C, Jones PW, Alldersea J, Fryer AA, Hoban PR and Strange RC (1998) Association of glutathione S-transferase GSTM1 and GSTT1 null genotypes with clinical outcome in epithelial ovarian cancer. *Clin Cancer Res* (In press)

### 1.9 Polymorphisms in the p53 family of tumour suppressor genes and suceptibility to breast cancer

*KL Søndergaard[1], D Wheatley[2], S Kelly[2] and AG Demaine[1]*
[1]Department of Molecular Medicine, Plymouth Postgraduate Medical School, Plymouth PL4 8AA, UK; [2]Oncology Dept., Derriford Hospital, Plymouth PL6 8DH, UK

p73, a novel homologue of the p53 tumour suppressor gene family, has recently been identified and mapped to chromosome 1p36, a region frequently deleted in several cancers. p73 has been demonstrated to be monoallelically expressed. An allelic polymorphism exists within its 5′ untranslated region consisting of a double nucleotide substitution (G→A) and (C→T) at position 4 and 14 of exon 2. We investigated the frequency of this genotype in the DNA extracted from peripheral blood samples of 75 patients with breast cancer and 100 sequential cord blood samples using PCR-RL. Out of the 75 patients, 28 (37.3%) were heterozygous for the C/T polymorphism and out of 100 controls, 33 (33%) also had the C/T polymorphism. The frequency of the C/T polymorphism was compared with tumour grade, size and node involvement and no significant correlation was found between clinical features and genotype. However, a non-significant trend was observed with the age of diagnosis. Twice as many patients diagnosed after 40 years of age had the GC monoallelic genotype (65.6%, 40 of 61) rather than the C/T polymorphism (34.4%, 21 of 61). Patients diagnosed before 40 years of age had a similar frequency of p73 genotypes. As half of all breast tumours harbour alterations in the p53 tumour suppressor gene, we also investigated the frequency of p53Pro/Arg polymorphisms in all of the samples. Interestingly, 53.2% (25 of 47) of the G/C p73 genotype was p53Pro/Arg heterozygous and 44.7% (21 of 47) was p53 Arg homozygous. In contrast, 39.3% (11 of 28) of the C/T p73 genotype was p53 Pro/Arg heterozygous and 60% (17 of 28) was p53Arg homozygous. It has recently been shown that individuals homozygous for p53Arg are 7 times more likely to develop human papillomavirus (HPV) associated cancer. An association may exist between p73 and p53Pro/Arg genotype in patients with breast cancer.

### 1.10 CDKN2A germline mutations in UK patients with multiple primary melanomas and familial melanoma

*RM MacKie, N Andrew, WG Lanyon and JM Connor*
Department of Dermatology and Medical Genetics, Glasgow University, Glasgow, UK

Approximately 5% of all melanoma patients have a family history of melanoma. We have investigated 16 families in the North of the UK and found that 6 of these have functionally damaging mutations of the CDKN2A (p. 16) gene. There are four different mutations observed. One family has an Arg 24 Pro substitution, one a stop codon at codon 44 of exon 1 and 4 have Met 53 Ile substitutions. The remaining ten members of multiple melanoma families do not appear to have CDKN2A mutations.

We have further investigated 17 patients with multiple primary melanomas and a negative family history after exhaustive questioning and examination. Two of these 17 also have CDKN2A mutations, one a Met 53 Ile substitution in exon 2 and one a G-T substitution at IVS2 + 1 splice donor site.

This is the first report from Europe of CDKN2A mutations in patients with multiple primary melanomas. There is one other report in the literature from Canada of patients with multiple primary melanomas and a negative family history having CDKN2A mutations.

Our observations to date suggest that CDKN2A germline mutations may be commoner than previously recognized. However it is also clear that two thirds of familial melanoma patients and a proportion of those with multiple primary melanomas may have mutations elsewhere. The search for other mutations continues.

### 2.1 Cytotoxicity of the non-steroidal anti-inflammatory drugs in human colonic cancer cells: a role for the polyamines?

*PB Low and HM Wallace*
Departments of Medicine & Therapeutics and Biomedical Sciences, University of Aberdeen, Foresterhill, Aberdeen AB25 2ZD, UK

Epidemiological studies have shown that regular use of non-steroidal anti-inflammatory drugs (NSAIDs) can decrease the incidence of colorectal cancer in man by as much as 50% (Rosenberg et al (1991) *J Natl Cancer Inst* **83**: 335). The mechanism is, however, unclear. It has been suggested that the chemopreventative effect is mediated through the prostaglandins with NSAIDs decreasing their production via inhibition of COX enzymes. Recently this theory has been questioned since, in cell culture, addition of prostaglandins did not reverse the effects of the NSAIDs on cell proliferation (Hanif et al (1996) *Biochem Pharmacol* **52**: 237). Another possibility is that NSAIDs interfere with polyamine metabolism which consequently affects cell growth. The polyamines, like the prostaglandins, are essential for growth, are found in high concentrations in many cancers (Wallace (1995) *Proc Nutr Soc* **55**: 351) and can regulate apoptosis in human cells (Lindsay and Wallace (1997) *Br J Pharmacol* Suppl.

© 1999 Cancer Research Campaign

**122**: 262P). In this study we have examined the effect of NSAIDs on cell growth and polyamine metabolism in a human colorectal cancer cell line (HT115). Salicylate, sulindac and naproxen all inhibited the growth of the cells with $IC_{50}$ values of approximately 7.5 mM, 0.75 mM and 3 mM respectively. Cytotoxicity was observed at higher doses. Polyamine content was decreased in cells treated with NSAIDs. Addition of exogenous spermidine resulted in the recovery of both cell growth and polyamine content. Therefore we suggest that the loss of polyamines induced by NSAIDs contributes significantly to the observed cytotoxicity and therefore the chemopreventative effects of these drugs.

### 2.2 Sulindac, TNFα signalling and apoptosis in intestinal epithelium

*B Ansari, A Kubba, K Atherton and FC Campbell*
Department of Surgery, University of Newcastle, Newcastle, UK

Sulindac sulphide may promote apoptosis and inhibit carcinogenesis through COX-2 independent mechanisms but relevant molecular pathways are unclear. TNF-α is an inflammatory cytokine which influences apoptosis through activation of NF kappa B (NF-κB). NF-κB induces transcription of an anti-apoptotic gene, IEX-1L. We test the hypothesis that sulindac sulphide promotes apoptosis in intestinal epithelium partly through inhibition TNFα-induced NF-κB activation.

The HCT116 colonic carcinoma and INT 407 benign human embryonic intestinal cell lines were transiently transfected with a NF-κB luciferase reporter construct, incorporating 3 synthetic copies of the NF-κB promotor upstream of transcription start site (3 enh-kb-CONA-luc) or control empty vector lacking kappa B sequences (CONA-luc). Cells were pre-incubated with or without sulindac sulphide (50–150 μM) then stimulated with 10 ng $ml^{-1}$ recombinant human TNFα. Apoptosis was assessed by acridine orange staining.

TNFα-stimulated NF-κB activation in transfected HCT116 and INT 407 cells, by up to fourfold. Sulindac sulphide induced dose dependent apoptosis in both cell lines, of up to 90% at 125 μM. Sulindac sulphide inhibited TNFα-stimulated NF-κB activation in both cell lines.

Sulindac sulphide may retard carcinogenesis in intestinal epithelium, partly by inhibition of TNFα induced NF-κB activation and consequent promotion of apoptosis.

### 2.3 Effect of soy or rye on colon tumours in AOM-treated rats

*MJ Davies[1], EA Bowey[1], H Adlercreutz[3], IR Rowland[1] and PC Rumsby[1]*
[1]BIBRA International, Carshalton, Surrey SM5 4DS, UK; [2]University of Helsinki, Meilahti Hospital, Helsinki, Finland

The value of a diet high in plant material in the prevention of cancer is apparent from epidemiological studies. Much current interest has focused on the possible role of a group of plant phenolic compounds with oestrogenic-like activity (phyto-oestrogens). In this study we have investigated the effects of isoflavones derived from soy and lignans from rye bran on the development colon tumours in F-344 rats treated with the carcinogen, azoxymethane.

Male F-344 rats were fed a carefully-balanced high fat diet based on isoflavone-free soy protein or the same diet with isoflavones (IF) or rye bran (RB). After 2 weeks on the diet, the rats received two injections of azoxymethane (AOM, 15 mg $kg^{-1}$) given 1 week apart. Animals were humanely killed after 12 or 31 weeks on the diet and the colons examined for aberrant crypt foci (ACE), the number of crypts per focus and the presence of tumours.

Analysis of the total number of ACF at 12 weeks revealed no differences between any of the diets. The number of small ACF (< 4 crypts/focus) was increased in the IF-containing group while the RB group showed a decrease in the number of larger ACF (> 6 crypts/focus). There were no differences in the small numbers of ACF which remained at 31 weeks. However, the number of tumours found in the colon at 31 weeks was significantly decreased in the RB group (mean tumour/rat: control 1.36, RB 0.166) while the IF group showed no change (mean tumour/rat, 1.38).

In conclusion, the supplementation of rye bran containing lignans was highly protective against AOM-induced tumours in the rat colon and this may involve the retardation of progression of early aberrant crypts although the mechanism of action requires further study. Soy isoflavones do not appear to offer any protection against AOM-induced colon cancer in this model.

This study was funded by EU FAIR Programme (Contract number 95/0894) and the World Cancer Research Fund.

### 2.4 Inhibition of leukaemia growth and apoptosis induced by phenethyl isothiocyanate and cysteine conjugate

*K Xu and PJ Thornalley*
Department of Biological Science, Essex University, Colchester CO4 3SQ, UK

Phenethyl isothiocyanate (PEITC) and its cysteine conjugate (PETC-Cys) have cancer chemopreventive activity in vivo. PEITC and PETC-Cys inhibited the growth of human leukaemia 60 (HL60) cells in serum-free medium in vitro – the $GC_{50}$ values were $0.84 \pm 0.01$ μM and $0.96 \pm 0.02$ μM respectively. The anti-proliferative activity of both compounds decreased with addition of increasing percentage of serum in the culture medium but the effect was more marked for PETC-Cys than for PEITC: with 25% serum, the $GC_{50}$ values were $4.57 \pm 0.21$ μM and $8.05 \pm 0.20$ μM respectively. Both compounds had much weaker effects on concanavalin A-stimulated proliferating peripheral human lymphocytes: the $TC_{50}$ values were $53.1 \pm 4.3$ and $90.8 \pm 2.7$ μM respectively. The inhibition of HL60 cell growth was characterized by a rapid interaction of PEITC with the cells in the first hour of culture – or exposure to the PEITC liberated from the cysteine conjugate PETC-Cys in the initial 3 h of culture. This interaction induced commitment to apoptosis which developed in the initial 24 h. Induction of apoptosis was judged by flow cytometry of cells stained with propidium iodide and by fragment ladders in Agarose gel electrophoresis of cellular DNA. Inhibition of macromolecular synthesis was also an early feature of the commitment to cell death. There was an increase of caspase-3 activity – determined by fluorimetric measurement of hydrolysis of the synthetic caspase-3 substrate *N*-acetyl-Asp-Glu-Val-Asp-7-amino-4-methylcoumarin but this was probably a marker rather than an obligatory mediating factor of isothiocyanate-induced apoptosis since the specific caspase-3 inhibitor *N*-acetyl-Asp-Glu-Val-Asp-aldehyde had no effect. The involvement of other caspases in isothiocyanate-

 © 1999 Cancer Research Campaign induced apoptosis was suggested, however, by inhibition of apoptosis with the non-selective caspase inhibitor N-benzyloxycarbonyl-Val-Ala-Asp(OMe)-fluoromethylketone. These effects may contribute to inhibition of pre-clinical tumour growth in chemopreventive activity.

### 2.5The chemopreventive agent curcumin suppresses cyclooxygenase-2 expression in colon cells by inhibiting the NIK/IκB kinase activation of NF-κB

*SM Plummer[1], KA Holloway[2], RJL Munks[1], MM Manson[1], A Kaptein[3], S Farrow[3] and A Hubbard[2]*
[1]MRC Toxicology Unit, [2]Centre for Mechanisms of Human Toxicity, University of Leicester, Leicester LE1 9HN, UK; [3]Glaxo Wellcome Research and Development, Stevenage, Hertfordshire SG1 2NY, UK

Curcumin, a chemical component of the curry spice turmeric, prevents colon cancer in animal models of this disease. We have previously shown that curcumin inhibits TNFα- and fecapentane-12-mediated induction of COX2 mRNA, NF-κB-DNA binding, in a normal colon epithelial cell line (HCEC) and NF-κB dependent transactivation in SW480 colon carcinoma cells (Holloway KA et al (1998) *Br J Cancer* **78**: 157). Since NF-κB is important in the regulation of the COX2 gene by inflammatory cytokines these data indicate that curcumin may inhibit COX2 induction in part via inhibition of NF-κB. Activation of NF-κB is modulated by a kinase signalling complex which contains NF-κB inducing kinase (NIK) and the IκB kinases (IKK) α and β. Phosphorylation of IκB by IKKα/β targets the protein for degradation via the proteosome which releases NF-kB into the nucleus to activate transcription. To determine whether curcumin would inhibit the degradation of the NF-κB sequestering protein IκB, we performed Western blot analysis with IκB-specific antibodies. Curcumin completely blocked the degradation of IκB caused by TNFα (10 ng $ml^{-1}$) in HCEC cells. To test the possibility that curcumin acts through inhibition of the NIK/IKK complex, we measured its ability to inhibit NF-κB-mediated alkaline phosphatase reporter gene activity (in the p4NFκB construct), induced either by exposure to TNFα (10 ng $ml^{-1}$) or by overexpression of the NIK kinase, following transfection of a NIK expression construct (pcDNA3-NIK). These experiments were carried out in a model cell system HEK293, circumventing problems associated with extremely poor transfection efficiency in HCEC cells. Curcumin (20 μM) inhibited TNFα-mediated induction of p4NFκB alkaline phosphatase activity by 54 ± 12% (s.d.) and a similar inhibition (49 ± 5%) was observed following induction of reporter gene activity by overexpression of NIK, suggesting curcumin acts via inhibition of the NIK kinase and/or signalling kinases downstream of NIK, namely IKKα and/or β.

### ACKNOWLEDGEMENT

We thank Dr Andrea Pfeifer, Nestle Research Center, for providing the HCEC cells.

### 2.6 Does rice contain cancer chemopreventive agents?

*A Dinh[1], T Kokubun[2], A Hudson[1], M Simmonds[2] and A Gescher[1]*
[1]MRC Toxicology Unit, University of Leicester, Leicester LE1 9HN, UK; [2]Jodrell Laboratory, Royal Botanic Gardens, Kew, Richmond, Surrey TW9 3AB, UK

There is growing public awareness of the potential beneficial effects on health of diet constituents. Whilst certain foods, such as soya beans and cruciferous vegetables, are thought to contain cancer chemopreventive agents, relatively little is known whether staple components of the human diet, such as rice (*Oryza sativa*, Gramineae), contain chemopreventive substances. We have investigated the growth-inhibitory properties (anticlonogenic activity) in SW 480 human colon cancer cells of extracts of cooked rice. Two commercially available rice varieties were studied, a highly refined (polished) white variety (var. *japonica*) and a brown variety (var. *javanica*). Rice grains (1.5 kg) were cooked in water and homogenized to a slightly viscous paste, which was extracted successively with diethyl ether, ethyl acetate and aqueous methanol (MeOH, 60%). Residues of extracts after filtration and concentration were analysed by HPLC ($C_{18}$ column, mobile phase: aqueous MeOH 40% to 100%, acetic acid 2%; UV detection 260 nm). Ethyl acetate extracts of the brown variety ('*jav* Etac') contained polyphenols and flavonoids, whilst such compounds could not be detected in extracts of the refined variety. '*jav* Etac' was fractionated (Amberlite XAD-2) into 10 fractions. Three of these fractions, when tested at 50 μg $ml^{-1}$, decreased the clonogenicity of the cells by more than 50% of controls. The active fractions were further fractionated by semi-preparative HPLC. Constituents of these subfractions were tentatively identified by co-chromatography as ferulic acid, p-methoxycinnamic acid (MCA) and several flavonoids. When tested separately, ferulic acid and MCA at 0.2 mM failed to affect cell clonogenicity, whereas the phenol gallic acid displayed an $IC_{50}$ value of 7 μM. The results suggest that the bran of brown varieties of rice may contain growth inhibitory agents of low molecular weight, the nature of which needs identification.

Supported by the World Cancer Research Fund.

### 2.7 Mechanisms of action of the dietary chemopreventive agent genistein

*EA Hudson, L Howells, P Chapman and MM Manson*
MRC Toxicology Unit, Hodgkin Building, Leicester University, Leicester LE1 9HN, UK

Epidemiological evidence has shown a potentially chemoprotective effect of Asian diets, which are high in soy products, when compared to Western diets. The isoflavanoid genistein, identified as an active component of soy, has been shown to exert antitumour activity in a range of rodent models. Several possible mechanisms of action have been proposed, including inhibition of tyrosine kinase, modulation of oestrogen receptor (ER) signalling, and inhibition of ornithine decarboxylase (ODC) activity.

© 1999 Cancer Research Campaign

We have investigated the effect of genistein on the growth and ornithine decarboxylase activity of a panel of human breast (HBL 100 ER+ve immortalized, MCF7 ER+ve tumour and MDA468 ER–ve tumour) and colon (HCEC immortalized, HT29 and SW480 tumour) cell lines. Growth curves were performed for each of the cell lines treated with genistein at 0, 10, 25, 40, 50 and 75 μM for 10 days. ODC activity in crude cell extracts was determined by measurement of $^{14}CO_2$ released from labelled substrate.

Genistein inhibited the growth of all six lines in a time and dose-dependent manner, the breast cells being more sensitive than the colon lines. The effect of genistein treatment on ODC activity varied with cell type. After 5-h treatment, ODC activity appeared to be increased in a dose-dependent manner in the HBL100 and SW480 cell lines (×3 and ×2 fold respectively at 75 μM), but decreased in the MDA468 cells (40% control values at 40 μM). ODC activity was not altered in the other cell lines, and at this time point, modulation of ODC activity did not correlate with the effect of genistein on cell growth. Preliminary results obtained with the SW480, MCF7, HBL100 and MDA468 lines after 24-h treatment showed a dose-dependent decrease in activity, suggesting that the effect of genistein on ODC activity is time-dependent, and may correlate more closely with the effect on growth at later time points.

The significance of the initial increase in ODC activity in the HBL 100 and SW 480 cells, and the relationship between inhibition of ODC activity and cell growth is currently under further investigation.

### 2.8 Evidence for autoproteolysis of substrate-inactivated *O*[6]-methylguanine-DNA methyltransferase following DNA repair

*GN Major[1,2], GB Notarianni[1], SJ Ross[1,3], MC Case[1,2,4], JA Brannigan[5], DI Roper[5], PM Potter [6], TP Brent[6] and PCE Moody[7]*

[1]Medical Molecular Biology Group (Department of Medicine), [2]Centre for Liver Research, and Departments of [3]Biochemistry and Genetics, and [4]Pathology, Medical School, University of Newcastle upon Tyne, NE2 4HH, UK; [5]Protein Structure Research Laboratory, Department of Chemistry, University of York, YO1 5DD, UK; [6]Department of Molecular Pharmacology, St. Jude Children's Research Hospital, Memphis, TN 38105–2794, USA; [7]Department of Biochemistry, University of Leicester LE1 7RH, UK

The anti-carcinogenic DNA repair and drug resistance enzyme, *O*[6]-methylguanine-DNA methyltransferase (MGMT), unusually functions in a single catalytic cycle. MGMT removes the methyl group from the *O*[6] atom of guanine, thereby restoring native guanine in DNA, and transfers the methyl group to itself at a specific cysteine residue. This automethylation inactivates the enzyme, which is not regenerated. That cells do not appear to demethylate and, hence, recycle alkylated MGMT as the active enzyme may be explained by others and our past findings of rapid degradation of the alkylated enzyme via the ubiquitin pathway. The present study arose from a potentially related observation, namely, our identification of a eukaryote thiol protease (active site histidine) motif in human MGMT (Collier *et al* (1996) *J Hepatol* **25**: 158–165). The presence of this motif raises the possibilities of MGMT having an intrinsic protease function and autoprotease activity that may be involved in enzyme degradation.

Here, we found that apparently homogeneous preparations (by several criteria, including their attaining theoretical maximum specific activity of ~ 46 nmol $mg^{-1}$ protein, and yielding clean N-terminal amino acid sequence) of recombinant human MGMT were able to undergo a time- and concentration-dependent degradation following repair of *O*[6]-methylguanine-DNA, that may be autoproteolytic in mechanism. This was observed for rMGMT prepared in two different ways: from a maltose binding protein (MBP)-MGMT fusion protein, following specific proteolytic cleavage of MBP from MGMT, and from unmodified human MGMT, over-expressed in *E. coli*. Putative methylated MGMT autoprotease activity was prevented by the thiol protease-specific inhibitor E64, and in an inhibitor concentration-dependent manner. The mixed-specificity thiol/serine protease inhibitor, leupeptin, similarly inhibited methylated MGMT degradation. Methylated MGMT autoproteolysis was also inhibited in the presence of excess native MGMT, indicating that (i) non-methylated enzyme is without autoprotease activity, (ii) protease activity appears upon DNA repair, (iii) the active enzyme is a potential substrate for proteolysis, and (iv) any contaminating protease with the ability to degrade MGMT would have to be highly specific, requiring activation by repair substrate *O*[6]-methylguanine-DNA. Highly purified MBP-MGMT was found to have DNA repair activity, but did not undergo degradation following automethylation, suggesting that MBP at the N-terminus of MGMT may have prevented enzyme autoproteolysis. It also provides further evidence that autoproteolysis seen with methylated rMGMT did not occur as a result of contaminating protease action.

Overall, we interpret these findings to be compelling evidence for MGMT having a catalytic site for proteolysis, and for it being able to undergo autoproteolytic modification following DNA repair.

### 2.9 Mouse models of DNA repair deficiencies

*D Barnes[1], A Klungland[2], H Nilsen[3] and T Lindahl[1]*

[1]ICRF, Clare Hall Laboratories, South Mimms, Herts EN6 3LD, UK; [2]The National Hospital, Oslo, Norway, [3]University for Science & Technology, Trondheim, Norway

DNA repair systems in human cells are targeted against three main types of lesions: (i) endogenous DNA damage generated by intracellular hydrolysis, active oxygen, and reactive cellular metabolites; (ii) mismatched nucleotides introduced by errors in DNA replication; (iii) pyrimidine lesions resulting from cellular exposure to ultraviolet light. Most DNA damage in normal proliferating cells is due to endogenous attack, and this unavoidable but potentially mutagenic and carcinogenic DNA damage is removed by highly effective repair mechanisms. The most important of these is base excision-repair. Two alternative strategies serve to correct an altered base; in the major pathway DNA polymerase beta replaces a single nucleotide, while a minor pathway involves replacement of several nucleotides in a FEN1- and PCNA-dependent reaction. We have reconstituted both branches of base excision-repair with purified proteins and synthetic DNA substrates (Kubota et al (1996) *EMBO J* **15**: 6662; Klungland and Lindahl (1997) *EMBO J* **16**: 3341).

Two major forms of endogenous DNA damage are (i) hydrolytic deamination of cytosine to uracil and (ii) oxidation of guanine to 8-hydroxyguanine (8-oxoG); both are mutagenic lesions, promoting GC to AT transitions and GC to TA transversions respectively. Uracil in DNA is removed by uracil-DNA glycosylase (UDG); bacterial and yeast mutants are viable but exhibit an increased spontaneous mutation frequency. Our group and others

 © 1999 Cancer Research Campaign have cloned human cDNAs encoding another DNA glycosylase able to remove 8-oxoG opposite C (OGG1) and the cognate gene has been mapped to 3p25, a locus often deleted in lung tumours (Roldan-Arjona et al (1997) *Proc Natl Acad Sci USA* **94**: 8016). We have generated viable mouse models deficient in either UDG or OGG1. Analysis of the mouse phenotypes with respect to spontaneous mutation frequency and tumour incidence will help to evaluate the contribution of the UDG and OGG1 enzymes to the maintenance of genetic stability, and the prevention of carcinogenesis.

We have also generated a knockout mouse model for DNA ligase IV, which is involved in DNA double-strand break repair and V(D)J recombination; DNA ligase IV null mutation results in embryonic lethality and the effects of the enzyme deficiency have been further investigated in mouse embryonic fibroblast cell lines.

### 2.10 Tumour targeting of anticancer drugs and oligonucleotides using polymeric carriers

*R Duncan, YN Sat, R Satchi, E Gianasi, N Malik and S Richardson*
Centre for Polymer Therapeutics, School of Pharmacy, 29–39 Brunswick Square, London WC1N 1AX, UK

N-(2-Hydroxypropyl) methacrylamide (HPMA) copolymer-doxorubicin (PK1) (Vasey P et al, (1998) *Clin Cancer Res*, (in press)), HPMA copolymer-platinate (AP5070) (Gianasi E et al. (1998) *Br J Cancer* **78**: 156) and dendrimer-platinates (Malik N and Duncan R (1997) *Proc Intl Symp Control Rel Bioact Mater* **24**: 527) all show ability to localize selectively in solid tumour tissue giving rise to significantly higher (> 70-fold) drug levels than can be achieved by administration of free drug. This passive targeting has been termed enhanced permeability and retention (EPR) effect Matsumura Y and Maeda H (1986) *Cancer Res* **46**: 6387) and it can also be used to target combination therapy, e.g. PDEPT (Satchi R and Duncan R (1998) *Br J Cancer* **78**: 149) and used for delivery non-viral vectors for DNA delivery.

Using s.c. B16F10 murine melanoma as a standardized screening system we have compared the whole body pharmacokinetics of the above mentioned conjugates and also polyamidoamines (PAAs) developed as endosomolytic polymers to facilitate intracytoplasmic access of oligonucleotide delivery systems. PK1 displayed tumour uptake in the range 1–14 % dose $g^{-1}$ with maximum uptake within 1 h. Due to plasma protein interaction the HPMA copolymer platinate had a longer circulation time and as a result showed progressive tumour accumulation over 24–48 h, maximum uptake was in the range 8–13 % dose $g^{-1}$. The hyperbranched dendrimer-platinate behaved more like PK1 with maximum tumour uptake at earlier time points, but the extent of capture was less (0.5–4% $g^{-1}$) and rate of egress from the tumour was faster. PAAs accumulated progressively in B16F10 after i.v. administration over 5 h. After administration of PK1 there is a lag phase (30–60 min) before free doxorubicin appears. This is consistent with intracellular drug release following pinocytic uptake of the conjugate. Administration of HPMA copolymer-cathepsin B 5 h after administration of PK1 (as a model to study the PDEPT concept) led to immediate release of doxorubicin indicating the possibility to modulate drug release extracellularly. This library of information regarding a variety of polymeric vectors sheds light on the factors important for tailor-making improved polymeric drugs and DNA vectors.

### REFERENCES

Vasey P et al (1998) *Clin Cancer Res* (in press)
Gianasi E et al (1998) *Br J Cancer* **78**: 156
Malik, N and Duncan R (1997) *Proc Intl Symp Control Rel Bioact Mater* **24**: 527
Matsumura Y and Maeda H (1986) *Cancer Res* **46**: 6387
Satchi R and Duncan R (1998) *Br J Cancer* **78**: 149

### P1 The generation of antibody fragments to the DNA adduct 8-oxoguanine

*CH Seedhouse[1], JH Hendry[2], GP Margison[2] and MJ Embleton[1]*
CRC Sections of [1]Cell and Tumour Biology and [2]Genome Damage and Repair, Paterson Institute for Cancer Research, Christie Hospital NHS Trust, Wilmslow Road, Manchester M20 9BX, UK

Oxidative damage to cellular DNA has been implicated in various age-related degenerative diseases, including cancer and heart disease. We describe a novel approach based on the use of phage display libraries to generate recombinant human antibody fragments against the DNA adduct 8-oxoguanine, which is an abundant mutagenic base product of oxidative damage. Several monoclonal single chain Fv (scFv) antibodies have been generated, and characterization in a quantitative ELISA system has shown one of these antibodies to have a lower limit of detection of $2.5 \times 10^{-13}$ mol 8-oxoguanine in a synthetic oligonucleotide. The antibody also binds to 8-oxoguanine lesions introduced into plasmid DNA by treatment with methylene blue and white light. The extent of antibody binding to treated DNA was proportional to the concentration of methylene blue and therefore to the level of 8-oxoguanine. The antibody also inhibits the release of 8-oxoguanine from 8-oxoguanine-containing oligonucleotides by the *E. coli* formamidopyrimidine-DNA glycosylase as determined using an in vitro system. Further experiments are in progress to exploit the ability of the antibody fragment to detect the adduct, and its biological activity in relation to DNA repair.

This work is supported by the Cancer Research Campaign.

### P2 *N*-desmethyltamoxifen forms DNA adducts in treated rats

*K Brown, R Heydon, R Jukes, INH White and EA Martin*
MRC Toxicology Unit, University of Leicester, Leicester LE1 9HN, UK

The anti-oestrogen tamoxifen is currently undergoing evaluation as a chemopreventive agent in women at an increased risk of developing breast cancer. However, tamoxifen treatment is associated with an increased incidence of endometrial cancer, and administration to rats causes hepatic tumours. Tamoxifen requires metabolic activation to electrophilic species before it can react with DNA, forming adducts detectable using the $^{32}P$-post-labelling assay. Using HPLC with on-line radiochemical detection $^{32}P$-post-labelled tamoxifen DNA adducts can be separated into at least 12 individual peaks (Martin EA *et al* (1998) *Carcinogenesis* **19**: 1061–1069). The major metabolites of tamoxifen in both rat and human liver are *N*-desmethyltamoxifen and 4-hydroxytamoxifen. However, the two major DNA adducts detected in the livers of tamoxifen-dosed rats arise as a consequence of $\alpha$-hydroxylation, which is a minor metabolic pathway. These adducts contain tamoxifen linked via the $\alpha$-carbon to the exocyclic amino group of deoxyguanosine. The relative importance of each adduct in the carcinogenic process is currently unknown and it is possible

© 1999 Cancer Research Campaign that more minor adducts arising from *N*-desmethyltamoxifen or 4-hydroxytamoxifen may be important.

In order to identify which of the in vivo adducts contain an *N*-demethylated tamoxifen moiety, rats were dosed with *N*-desmethyltamoxifen (40 mg $kg^{-1}$ i.p. daily) for 4 days. This metabolite was synthesized from tamoxifen by a novel route involving formation of a demethylated trichloroethyl carbamate derivative followed by reduction with zinc in acetic acid. The liver DNA was extracted and subjected to $^{32}P$-post-labelling and HPLC separation. Two peaks were observed. The largest peak co-eluted with one of the major rat liver adducts described above. The second peak co-eluted with a minor, more polar tamoxifen adduct. LC-MS analysis of the liver extracts showed the absence of tamoxifen, confirming that the adducts were derived from *N*-desmethyltamoxifen. The levels of adducts formed, 100 ± 33 $10^{-8}$ nucleotides, $n = 4$, was comparable to that seen when rats were dosed with tamoxifen itself (109 ± 36 adducts $10^{-8}$ nucleotides, $n = 3$). The finding that the main *N*-desmethyltamoxifen adduct co-elutes with one of the major tamoxifen adducts demonstrates that loss of a methyl group does not significantly affect chromatographic mobility in this HPLC system. This highlights the need to identify all the components of the main adduct peaks since it is likely that additional structurally different adducts may also elute at this time.

### P3 Modification of nucleotides and 2′-deoxyguanosine by nitrosated indoles results in deamination, depurination and the formation of a novel product oxanine via a transnitrosation mechanism

*LT Lucas[1], D Gatehouse[2] and DEG Shuker[1]*
[1]Biomonitoring Section, MRC Toxicology Unit, University of Leicester, Leicester, LE1 9HN, UK; [2]Genetic and Reproductive Toxicology, Glaxo Wellcome Research and Development, Ware, Herts, SG12 0DP, UK

Indoles are a major group of naturally occurring compounds with chemoprotective properties found in cruciferous vegetables. Synthetic 3-substituted indoles have shown potential as pharmaceutical agents (Sinha and Jain, 1994) Indoles can be readily nitrosated to give the corresponding N-nitrosoindoles and this pathway is of concern since there are endogenous sources of nitrite and nitrosating agents. Indole-3-acetonitrile, a precursor in Chinese cabbage, becomes mutagenic upon nitrite treatment (Wakabayashi et al, 1985) and has been shown to form DNA adducts in rat stomach (Yamashita et al, 1988). In this study, the reaction of a series of nitrosated indoles with 2′-deoxyguanosine-5′-monophosphate (dpG), 2′-deoxyadenosine-5′-monophophate (dpA) and 2′-deoxyguanosine (dGuo) was investigated. Indole-3-acetonitrile, indole-3-acetamide and indole-3-acetic acid methyl ester were all nitrosated and the products characterized by mass spectrometry, NMR and elemental analysis. Incubations between the nitrosated indole and nucleotide or nucleoside were carried out at neutral pH, employing a monophasic reaction system with acetonitrile as the co-solvent. Resulting reaction mixtures were analysed by high performance liquid chromatography, UV spectrophotometry and electrospray mass spectrometry. Reactions with all three N-nitrosoindoles gave rise to the same profile of nucleotide or nucleoside modifications with the products exhibiting a dose–response relationship. Depurination to guanine and adenine was evident, as was deamination coupled with depurination, resulting in xanthine and hypoxanthine as the major products. The formation of 2′-deoxyoxanosine-5′-monophosphate and the depurination product oxanine was observed for reactions with dpG, and oxanine was observed with dGuo. In addition, N-acetyl adducts were evident, the source of acetyl groups being the co-solvent. Analogous products were seen when incubations were completed with 3′-monophosphates. Denitrosation was the major decomposition pathway of the nitrosated indoles in this system. These findings combined with identical reaction products for all nitrosated indoles and a positive result in the Liebermann nitroso test at neutral pH suggests transnitrosation as the main reaction pathway. In conclusion, these results indicate that N-nitrosoindoles do not modify DNA through alkylation. Instead they appear to mimic a nitric oxide type chemistry and undergo N-N heterolytic cleavage to transfer the nitroso group to purine bases resulting in deamination, depurination and the formation of a novel product oxanine, all of which are potentially mutagenic events.

Support from Glaxo-Wellcome is gratefully acknowledged.

### REFERENCES

Sinha S and Jain S (1994) *Prog Drug Res* **42**: 59–75
Wakabayashi K, Nagao M, Ochiai M, Tahira T, Yamaizuma Z and Sugimura T (1985) *Mutat Res* **143**: 17–21
Yamashita K, Wakabayashi K, Kitagawa Y, Nagao M and Sugimura T (1988) *Carcinogenesis* **9**: 1905–1907

### P4 Detection of malondialdehyde-deoxyguanosine in white blood cells of volunteers on standardized diets

*C Leuratti[1], R Singh[1], E Deag[1], R Hughes[2], S Bingham[2], E Griech[1], LJ Marnett[3] and DEG Shuker[1]*
[1]MRC Toxicology Unit, University of Leicester, UK; [2]Dunn Nutrition Centre, Cambridge, UK; [3]Vanderbilt University, Nashville, TN, USA

DNA adducts can be formed upon exposure of DNA to both exogenous and endogenous genotoxic agents. Malondialdehyde (MDA) is an endogenous product of lipid peroxidation and prostaglandin biosynthesis. The main adduct formed with DNA, MDA-deoxyguanosine ($M_1$-dG) has been detected in human tissues and leucocytes. The aim of our work is to conduct a series of studies in both volunteers and free-living populations in order to examine whether diet modulates $M_1$-dG formation in humans. Dietary studies are being carried out with male volunteers housed in the metabolic suite of the Dunn Nutrition Centre where diet can be manipulated to desired levels. Initial results obtained by HPLC/$^{32}P$-post-labelling (Leuratti et al (1998) *Toxicol Lett* **95** (Suppl 1) 189) showed a tenfold increase in $M_1$-dG levels in white blood cell (WBC) DNA of two individuals on a red meat diet compared to a free diet period. Introduction of vegetables and black tea significantly reduced those levels. More recent results using an immunoslot blot assay (Leuratti et al (1998) *Carcinogenesis* **11**: in press) confirmed a possible effect of a red meat diet on $M_1$-dG adduct levels which increased from undetectable (free diet) to 10.05 (red meat) adducts per $10^7$ normal deoxynucleosides. Presently, DNA samples from WBC of volunteers maintained on a high meat diet for 40 days are being analysed in order to examine possible time effects on $M_1$-dG levels. Blood samples are being collected at 5-day-time intervals. Results obtained so far showed $M_1$-dG levels ranging from 2.4 to 10.8 adducts per $10^7$ deoxynucleosides.

 © 1999 Cancer Research Campaign

In a second part of our work, $M_1$-dG levels will be measured in free-living populations for which samples have been collected and dietary information are available through EPIC (European Prospective Investigation on Cancer). Before carrying out analysis on these samples, a detailed knowledge about the effect of long term storage of samples on the stability of biomarkers of DNA damage is required. In our laboratory, studies are ongoing on the stability of $M_1$-dG in blood samples stored for up to 5 years.

This study is funded by UK MAFF, contract no. FS1716 and FS1735.

### P5 Risk assessment for liver cancer in women taking tamoxifen

*P Carthew, EA Martin, LL Smith, RE Edwards, RT Heydon and BM Nolan*
MRC Toxicology Unit, Hodgkin Building, University of Leicester, Leicester LE1 9HN, UK

Tamoxifen is a cumulative genotoxic hepatocarcinogen in the rat (Carthew et al, 1995*a*) and, although most of the human carcinogenicity associated with tamoxifen use has involved the uterus (Van Leeuwen et al, 1994), it is still unclear whether tamoxifen presents a possible long-term hepatocarcinogenic potential in women. To clarify this possibility we have fed tamoxifen to rats for differing periods of time (1–12 weeks) to induce increasing amounts of DNA adducts in the liver. The dose–response relationship between tamoxifen-specific DNA adduct formation and the subsequent development of liver tumours over the next 2.5 years has been examined to determine the nature of the dose–response relationship. To increase the sensitivity of the bioassay groups of tamoxifen-exposed rats were also given phenobarbital in the drinking water to model a worst case scenario of liver cell proliferation and promote tumour formation (Carthew et al, 1995*b*). A dose–response relationship was found for adduct levels and liver tumour formation and a no effect level (NOEL) for tamoxifen-induced liver tumours and DNA adducts was determined. The comparison of this NOEL to the available data for adduct levels in liver biopsies from women taking tamoxifen showed that a significant safety margin of approximately 100-fold exists between the adduct levels in rats at the no effect level for liver tumours and the levels of adducts found in women taking tamoxifen. This new method of risk assessment based on the correlation of levels of adducts known to be mechanistically related to subsequent cancer formation in rodents with levels of the same adducts found in man will be useful in further risk assessment for potential human carcinogens.

### REFERENCES

Carthew P et al (1995*a*) *Carcinogenesis* **16**: 1299
Carthew P et al (1995*b*) *Cancer Res* **55**: 544
Van Leeuwen FE et al (1994) *Lancet* **343**: 448

### P6 Ezrin regulates cell–cell adhesion and motility and associates with intercellular junctional proteins in cancer cells

*S Hiscox, RE Mansel and WG Jiang*
Department of Surgery, University of Wales College of Medicine, Cardiff CF4 4XN, UK

Ezrin is a member of the ERM family of proteins that are thought to function as cross-linkers between the plasma membrane and the action cytoskeleton. Ezrin has also been implicated as playing a role in intracellular signal transduction since both its tyrosine-phosphorylation and translocation occurs in response to growth factor receptor stimulation. Studies have shown that these proteins can directly associate with cell surface adhesion molecules including CD44 and ICAM2 and thus they may play a role in mediating cell–cell adhesion. The aim of this study was thus to investigate further the role of ezrin as a mediator of cell adhesion and metastatic behaviour in colorectal cancer cells, HRT18 and HT115.

Following experimental inhibition of ezrin expression within these cells using anti-sense oligonucleotides, time-lapse video microscopy demonstrated that both cell types showed a loss in intercellular adhesions. HT115 cells, which lack E-cadherin, were quickly dissociated following ezrin inhibition, whereas in HRT18 cells, which express E-cadherin and form tightly-packed colonies, there was both a marked formation of intercellular gaps within these colonies together with a movement of cells at the colony edge away from the main colony body. These changes were confirmed with transmission electron microscopy studies, which showed a marked degeneration of the cell–cell junctions within these cells after anti-sense treatment.

Induction of loss of ezrin expression with anti-sense oligonucleotide also enhanced the motile and invasive capabilities of these cells particularly following stimulation with HGF/SF, a potent promoter of metastatic cell behavior, as shown by a phagokinetic colloidal gold assay and basement membrane invasion assays respectively.

Immunoprecipitation of ezrin from the cell lysates revealed that in HRT18 cells, both β-catenin and E-cadherin co-precipitated along with ezrin, whereas in HT115 cells which lacked E-cadherin expression, ezrin was associated with the desmosomal cadherin, Dsg.

In conclusion, these results show that removal of ezrin from colon cancer cells results in dissociation of cell colonies together with an enhancement of both their motile and invasive capabilities. These effects are likely to be the consequence of loss of cell–cell adhesion mediated by cadherin complexes, due to absence of ezrin. These results thus implicate ezrin as a regulator of cadherin-mediated adhesion and thus as a potential suppressor of tumour cell motility and invasion.

### P7Induction of apoptosis in the hepatoma-derived cellline, PL[illegible]

*A Redfern and WG Jiang*
Department of Surgery, University of Wales College of Medicine, Cardiff CF4 4XN, UK

Essential fatty acids (EFAs) cannot be synthesized by the human body and must therefore be obtained from the diet. In recent years, certain EFAs, including γ-linolenic acid (GLA), have been shown to be selectively toxic to tumour cells. This study was designed to investigate the mode of cell death in the cell line PLC/PRF/5 following treatment with GLA.

Extensive DNA cleavage, a characteristic feature of apoptosis, was assessed by flow cytometry using a method based on the extraction of low molecular weight DNA prior to cell staining. This results in apoptotic cells containing a reduced DNA content and therefore can be recognized as cells with low DNA stainability which are seen as a sub G1/G0 peak. In addition, Western blotting

© 1999 Cancer Research Campaign 

was carried out on total cell lysates from cells treated with a range of GLA concentrations (0–100 μM, 24 and 48 h). The membranes were then probed with antibodies against Bcl-2 family members to determine whether any changes occurred in their expression following GLA treatment.

Flow cytometry data indicated that high levels of GLA (200 and 300 μM) induced apoptosis after 24 h of treatment. The percentage of events below G1/G0 was, on average, 7.0% and 14.8% for 200 μM and 300 μM GLA, respectively, compared to a control value of 2.8%. However, by 48 h apoptosis was induced in cells treated with 100 μM GLA (on average, 5.3% compared to a control value of 3.5%). Analysis of the cell cycle showed, at higher levels of GLA, a slight trend towards a reduction in the population of cells in G2/M and a concomitant increase in the population of cells in S phase. This may suggest an arrest of the cell cycle in S phase. Staining-treated cells with Hoechst 33528 confirmed the presence of an increased number of apoptotic cells in samples treated with GLA compared to control samples. Western blotting indicated that Bcl-Xs, a Bcl-2 family member was present at low levels that increased, over 24 h, with increasing GLA concentration. However, no detectable level of Bcl-2 protein or its antagonist Bax was observed. Additionally, Bag-1, a Bcl-2 binding protein was detected, but no changes in expression were observed.

In conclusion, these data show that GLA induces apoptosis in the hepatoma-derived cell line PLC/PRF/5, an effect, at least in part, associated with changes in Bcl-2 family members, such as Bcl-Xs.

### P8 Effects of flavonoids on primary oral cultures: enhanced sensitivity of tumour cells to growth inhibition by morin

*J Brown[1], F McGregor[1], J O'Prey[1], E Wagner[1], D Felix[2], D Soutar[3] and PR Harrison[1]*

[1]The Beatson Institute for Cancer Research, CRC Beatson Labs, Glasgow G61 1BD, UK, [2]Department of Oral Medicine, Dental School, Glasgow G2 3JZ, UK; [3]Department of Plastic Surgery, Canniesburn Hospital, Glasgow G61 1QL, UK

Flavonoids are chemopreventive in several chemically induced animal cancer models and may be one of the components of fruits and vegetables responsible for chemoprotective effects in humans. The effects of eight naturally occurring flavonoids were investigated on a panel of oral primary cultures derived from biopsies from normal oral mucosa and oral lesions at various stages of cancer progression, i.e. early dysplasias, late-stage tumours and metastases. Cells were treated with flavonoids for 3 days and $^3$H-thymidine incorporation was measured in the final 6 h. All the flavonoids inhibited oral cell growth with $IC_{50}$ values in the range 18–54 μM, but they did not show any stage specificity. However, although higher concentrations of morin were required to inhibit cell growth, it had significant tumour specificity ($IC_{50}$ for tumour and normal cultures 110 μM and 180 μM respectively). This change in specificity occurred at the dysplasia stage of cancer progression. Structure function comparisons with other flavonoids showed that the tumour-specific effect of morin required both the 2′ and 4′ hydroxyl groups. Further studies with quercetin and morin have shown that they inhibit the growth of oral carcinomas in the G2/M phase of the cell cycle, with no evidence of apoptosis at these growth inhibitory concentrations.

Flavonoids are known to inhibit various signalling enzymes involved in cell growth. We are presently testing candidate enzymes which may be inhibited by flavonoids and which may be activated during cancer progression. We have shown that the growth inhibitory effect of quercetin but not morin can be reversed by the addition of the lipoxgyenase metabolites 5-, 12- and 15-HETEs, suggesting that the lipoxgyenase signalling pathway is involved the growth inhibitory effects of quercetin. In addition, we have found that activation of the stress kinase pathway may be involved in the growth inhibitory effect of morin.

### P9 Cytochrome P450 CYP1B1 in breast cancer

*GI Murray[1], S Buchan[1], MCE McFadyen[1], ID Miller[1], S Payne[1] and WT Melvin[2]*

Departments of [1]Pathology, [2]Molecular and Cell Biology, University of Aberdeen, Foresterhill, Aberdeen AB25 2ZD, UK

Cytochrome P450 CYP1B1 is the only known member of a recently identified sub-family of the cytochrome P450 CYP1 gene family. We have previously shown increased expression of CYP1B1 in several types of human cancer. Human CYP1B1 expressed in yeast shows high specific activity towards the 4-hydroxylation of 17β-oestradiol converting it to 4-hydroxyoestradiol, while there is significant 4-hydroxylation of oestradiol in breast cancer. In this study we have developed monoclonal antibodies to human CYP1B1 and used these antibodies to investigate the expression of CYP1B1 by immunohistochemistry in a series of primary breast cancers. The monoclonal antibodies were generated using a synthetic peptide coupled to carried protein as the immunogen. Hybridoma clones were initially screened using peptide conjugate, and positive clones were then also tested for recognition of human CYP1B1, which had been expressed in lymphoblastoid cells. The monoclonal antibodies specifically recognized CYP1B1 and the antibodies did not recognize either expressed CYP1A1 or CYP1A2. CYP1B1 was not detected in several normal tissues including liver, lung, small intestine and kidney. The monoclonal antibodies were also tested by immunohistochemistry using sections of a breast cancer, which we have previously shown to contain a high level of CYP1B1. One of the antibodies was found to be effective by immunohistochemistry on formalin-fixed, wax-embedded tissue sections and was used in the subsequent immunohistochemical studies. The majority of breast cancers showed positive immunoreactivity for CYP1B1 and CYP1B1 was specifically localized to tumour cells. The presence of CYP1B1 in breast cancer cells is likely to contribute to their metabolism of oestradiol and CYP1B1 also provides a molecular target for anticancer drugs specifically activated by this form of P450.

This research has been supported by a grant from the Medical Research Council.

### P10 Expression of hypoxia-inducible factor-1α and vascular endothelial growth factor in glioblastoma multiforme

*KL Søndergaard[1], D Hilton[2] and AG Demaine[1]*

[1]Department of Molecular Medicine, Plymouth Postgraduate Medical School, Plymouth PL4 8AA, UK; [2]Department of Histopathology, Derriford Hospital, Plymouth PL6 8DH, UK

Hypoxia-inducible factor-1 alpha (HIF-1α) is a transcription factor that stabilizes wild-type p53 and is widely expressed in tissues under hypoxic conditions. HIF-1α transcriptionally activates

 *© 1999 Cancer Research Campaign*

vascular endothelial growth factor (VEGF) and is thought to play a critical role in the expression of genes involved in angiogenesis. Although VEGF is known to be up-regulated in many tumours including glioblastoma multiformes (GBM), at present little is known about the mode of expression of HIF-1α. The aim of this study was to investigate the expression of HIF-1α, as well as VEGF and p53, in 25 GBM samples using RT-PCR and immunocytochemistry. Out of the 25 tumour samples, 20 (80%) expressed HIF-1α, 21 of the 25 (84%) expressed VEGF and 16 of the 25 (64%) tumour samples displayed overexpression of p53 (> 20% labelling). Of the 20 HIF-1α-positive samples, 13 (65%) displayed overexpression of p53, with three of the five (60%) HIF-1α-negative tumours displaying overexpression of p53. Only one of the five (20%) HIF-1α-negative tumours was also VEGF-negative. VEGF expression varied from 1- to 100-fold (mean 25-fold) in all of the GBM tumour samples. All GBM tumour samples displayed similar degrees of necrosis and vascular proliferation. This study did not find a correlation between HIF-1α expression, VEGF expression, overexpression of p53, tumour vascularity or necrosis.

### P11 The profile of MHC antigen expression in normal and leukaemic B-cells in patients with choronic lymphocytic leukemia (CLL)

*AME Nouri, Z Entezami, M Macey and RTD Oliver*
Department of Medical Oncology, Royal London Trusts, London E1 1BB, UK

This study was set up to investigate the profile of MHC class I and class II antigens in CLL patients using flowcytometric analysis. In total 13 patients were used, most of whom have had no prior treatment.

The results can be summarized as follows:

1. All CD5-negative cells expressed class I and class II antigens. The per cent of cases positive for polymorphic HLA-Bw6, -Bw4, -B7, -A2 and -B27 antigens were 84, 46, 15, 23 and 7 respectively.
2. In all the cases, Vim (antibody-specific for fibroblast and used as a negative control) and transferrin receptor showed negativity on CD5-negative cells.
3. The ratios of class II antigens for CD5-positive/CD5-negative cells were more than one in all cases except three (cases 2, 3 and 6). Similar results were observed for 12 of 13 cases for monomorphic class I (except case no. 5) antigens, whilst in the case of polymorphic class I antigens all the values were above unity.
4. Similarly, in all cases, the ratios for CD71 were above unity.

These results clearly indicated that, unlike solid tumours where the loss of class I antigens (particularly those detected by polymorphic-specific antibodies) is a frequent event, in CLL leukaemic patients this is not the case. That is to say, the loss or abnormal expression of these antigens does not play an important role in CLL malignancy. The overexpression of class II antigens on CD5-positive compared with CD5-negative cells is interesting, and experiments are in progress to assess the significance of this observation.

### P12 Interferon α and its various functional facets in comparison with other cytokines in an in vitro setting

*AME Nouri, ST Zubairi and RTD Oliver*
Royal London Trusts, London, UK

The clinical efficacy of interferon alpha (IFN-α), and to a lesser extent other cytokines, is well established. Using various techniques the biological activities of recombinant IFN-α as well as IFN-γ and an in-house prepared mix of cytokines or active supernatant (AC) were investigated.

Results showed that:

1. Whilst both IFN-α and AC up-regulated MHC class I antigens, class II antigens were only induced by AC. These results were also demonstrable for intracellular cell adhesion molecule (ICAM-1). Thus, the mean ± s.d. of radiobinding values for seven tumour lines for untreated, IFN-α and IFN-γ-treated cells were 617 ± 406, 943 ± 471 ($P = 0.001$) and 702 ± 563 ($P > 0.05$) cpm respectively.
2. Both IFN-α and AC increased the killing activity of IL-2-activated mononuclear cells (LAK) cells by as much as 15%. In addition, pretreatment of tumour target cells with IFN-α increased their susceptibility to killing by as much as 25%.
3. IFN-α and AC showed direct cytotoxic effects on some tumour cell lines like Wil (a bladder line) by as much as 36%.
4. Combination of IFN-α and cisplatin showed additive suppressive effects on tumour lines. In addition, tumour cell lines exposed to AC and cisplatin showed that, whilst the expression of inducible molecules like class II was severely decreased, there was only a modest inhibition of class I, EGFr and p53 molecules.

The findings of this investigation demonstrated the capacity of IFN-α to increase the visibility of tumour cells to the immune system by increasing the expression of MHC class I antigens. The data also demonstrated that IFN-α acted at other levels contributing to its overall clinical efficacy.

### P13 The evolution of LOH on chromosome 17 during the progression to Barrett's adenocarcinoma

*J Garde[1], J Dunn[1], K Dolan[1], J Gosney[2], R Sutton[3] and JK Field[1]*
[1]Molecular Genetics and Oncology Group, Clinical Dental Sciences, [2]Department of Pathology, [3]Department of Surgery, The University of Liverpool, Liverpool L69 3BX, UK; [4]Department of Surgery, Thameside General Hospital, Fountain Street, Ashton Under-Lyne, Greater Manchester OL6 9RW, UK

We have previously identified 13 common, minimally deleted regions (MRs) on chromosome 17 in 12 Barrett's oesophageal adenocarcinoma (BOA) specimens using multiple precisely mapped microsatellite markers (Dunn et al (1999) *Oncogene*, in press). The aim of the present study has been to identify the earliest sites of loss on this chromosome that arise and persist during the progression to BOA. This has been undertaken by the analysis of multiple carefully micro-dissected tissue samples from each of five oesophagectomy specimens, several of which contained identifiable premalignant tissue. Our data demonstrate a step-wise accumulation of loss in each analysed specimen, consistent with a single clonal pathway in four specimens and several coexisting pathways in one specimen. Several clonal anomalies (loss preceding heterozygosity, and variable intra-sample degrees of loss at different markers) were also observed. Within extensively deleted regions of the tumour (seen in three specimens), small deletions were detected in earlier tissue, predominantly at the site of our identified MRs, and these losses were seen to expand and merge during the progression to BOA. Some clonal losses at MRs were first detected in histologically early tissue, including metaplastic columnar epithelium, squamous epithelium and dysplasia. Our results provide further support for many of the MRs we have previously identified, thereby adding to evidence of the existeance of multiple novel tumour suppressor genes on chromosome 17 involved in the development of BOA.

### P14 Heat shock protein (Hsp) expression in ascitic ovarian cancer cells and related resistance to cytotoxic killing by lymphocytes

*X Han, B Kelly, GD Wilbanks, O Devaja and K Raju*
Department of Obstetrics & Gynaecology, St Thomas Hospital, London, UK

It is well known that heat shock proteins (Hsps) protect cells from a number of injurious factors (Lindquist (1988) *Annu Rev Genet* **22**: 631). Our previous study showed that co-culture of ascitic ovarian cancer cells with IL-2-activated lymphocytes induced immunoresistance of the ovarian cancer cells to immunocytotoxicity (Papadoupoulos et al (1995) *Gynecol Oncol* **57**: 388). This implies that *hsp* expression may play an important role in the mechanisms leading to this form of immunoresistance. The aim of this study was firstly to investigate HSP expression within ovarian cancer cells in vivo in ascites and in vitro in co-culture, and secondly to observe if immunoresistance was related to HSP expression.

Tumour/lymphocyte co-culture was used to induce immunocytotoxic (ICT) stress to ovarian cancer cells isolated from malignant ascites. Immunocytochemistry (ICC) using a panel of antibodies against Hsp27, 60, 70 and 90 was employed to detect *hsp* expression. $^{51}$Chromium-release cytotoxicity assay was used to evaluate resistance/sensitivity of ovarian cancer cells to lymphocyte mediated-cytotoxicity.

Freshly isolated ovarian cancer cells from ascites exhibited expressions of Hsp60, Hsp70 and Hsp90 in a variable range as tested by ICC. After co-culture with activated lymphocytes. ICT-stressed ovarian tumour cells exhibited overexpression of Hsps, predominantly Hsp90, tested by ICC. In $^{51}$Chromium release cytotoxicity assays, we found that ICT stressed ovarian cancer cells, which expressed HSPs, were resistant to IL-2 activated lymphocyte-mediated cytotoxicity. We also noticed a non-MHC restricted feature of this resistance as autologous lymphocyte stressed-tumour cells resist allogenic lymphocyte-cytotoxicity.

Data from our experiments demonstrate that Hsps expression can be found in vivo within ascitic ovarian cancer cells and also can be induced by in vitro ICT-stress. Such expression may be involved in resistance of tumour to cytotoxicity mediated by immune-competent cells. The clinical significance of this study lies in the possibility of opening a new avenue in the immunotherapy of ovarian cancer with alteration of *Hsp* gene expression by anti-sense treatment to augment immunocytoxicity mediated by immune-competent cells.

### REFERENCES

Lindquist S (1988) The heat-shock proteins. *Annu Rev Genet* **22**: 631
Papadopoulos AJ, Han X et al (1995) Induction of immunocellular resistance to IL-2-activated lymphocytes within ovarian carcinoma cells. *Gynecol Oncol* **57**: 388

### P15 Modulation of P-glycoprotein expression in ribozyme-transfected multidrug resistant (MDR) lung tumour cells

*EL Lewis[1] and I Gibson[2]*
[1]University of East Anglia, School of Biological Sciences, Norwich NR4 7TJ, UK; [2]House of Commons, Westminster, UK

Overexpression of the *MDR1* gene and its translation product, the trans-membrane energy-dependent efflux pump (Pgp: 170 kDa), is one of the major mechanisms by which cancer cells develop the MDR phenotype. The up-regulation of Pgp results in the premature expulsion of a variety of amphiphilic cytotoxic drugs and is a major impediment to successful chemotherapy (reviewed by Germann (1996) *Eur J Cancer* **32A**: 927). Efforts to reverse the Pgp-based MDR phenotype have focused principally on agents which are competitive inhibitors of the Pgp pump, or gene targeting (Bouffard et al (1996) *Eur J Cancer* **32A**: 1010). In order to reduce Pgp levels in MDR small-cell lung cancer cells, NIH H69/LX4, we have constructed recombinants for the exogenous (pFORTH: Tabler and Tsagris (1991) *Gene* **108**: 175) and endogenous (pHBetaAprl-neo: Gunning et al (1987) *Proc Natl Acad Sci USA* **84**: 4831) synthesis of long-chain asymmetric hammerhead ribozymes (catalytic anti-sense RNAs) targeting the regions of *MDR1* mRNAs predicted to have a low folding potential (Sczakiel et al (1993) *Antisense Res Dev* **3**: 453) and/or conserved sequences (Lewis and Gibson (1997), *BACR 38th Annual Meeting*, Abstract P57: 33)

Ribozymes and substrates for in vitro analyses were transcribed from the recombinant pFORTH vector-encoded T7 or T3 promoter, respectively, using Ampliscribe (Cambio). The catalytic activity of the ribozymes were assessed by electro-separation of the cleavage products through denaturing gels (Separide, Life Technologies). Constitutive expression of a ribozymes directed to exons 20 and 21 *MDR1* mRNA sequences in stably- (500 $\mu g\ ml^{-1}$ G418 selection) and transiently-liposome-transfected (LipofectAmine, Life Technologies) NIH-H69/LX4 cells resulted in a significant reversal of their resistance profile. The relative toxicity of the Pgp substrate doxorubicin (PNNAG Assay), expressed as $LD_{50}$ was reduced to parental (NIH H69/P) levels. The amplitudes of inhibition of MDR1 mRNA relative to β-actin (slot blotting) in NIH H69/LX4 cells expressing the ribozyme transiently- or stably were 27–37% or 80% respectively, concomitant with modulation of Pgp expression (Western immunoblotting: mAb C219) and ~60–78% increase in drug accumulation. The phenotype of the control cells (untreated, LipofectAmine-treated or expressing the vector alone) was vitually unaltered.

This work is supported by The Big C Appeal, Mrs P Salter, Francesca Gunn Leukaemia Appeal and AICR.

 *© 1999 Cancer Research Campaign*

### P16 Synergistic action of Temozolomide and thioguanine on glioblastoma xenografts

*DC Sullivan and PF Swann*
Biochemistry and Molecular Biology, University College London, London WC1E 6BT, UK

It has been proposed that the cytotoxic action of thioguanine involves the following steps: (1) thioguanine is incorporated into DNA; (2) S-adenosylmethionine methylates it to form $S^6$-methylthioguanine; (3) recognition of base pairs containing $S^6$-methylthioguanine by proteins of the post-replicative mismatch repair pathway leads to cell death (Swann et al (1996) *Science* **273**: 1109). The mechanism of Temozolomide, a methylating agent being used for the treatment of brain tumours, exactly parallels that of thioguanine: it methylates guanine to form $O^6$-methylguanine, then base pairs containing this base are recognized by proteins of the post-replicative mismatch repair pathway. Temozolomide would also methylate the thioguanine so, if the mechanism proposed for thioguanine were correct, one would expect a strong synergism between the two drugs. To test a combination of thioguanine and Temozolomide, a globlastoma (U87MG) was transplanted into nude mice. Seven days later (a.v. vol of tumour about 200 $mm^3$) the mice were given thioguanine (i.p., 4 mg $kg^{-1}$ $day^{-1}$; 4 days). Eight hours after the fourth injection the mice were given 5 mg $kg^{-1}$ Temozolomide (i.p.). There was essentially no difference between the control group and groups treated with thioguanine alone, or with Temozolomide alone. In all three cases the tumours reached 1000 $mm^3$ between 7 and 8 days after beginning the thioguanine treatment (i.e. 14 days after transplant). However, the tumours in mice treated with both Temozolomide and thioguanine were strongly affected and did not reach 1000 $mm^3$ until 18 days (i.e. 25 days after transplant).

As Temozolomide produces $O^6$-methylguanine there is an inescapable chance that its use will cause cancer. Increasing the effectiveness of Temozolomide by inhibiting $O^6$-alkylguanine-DNA-alkyltransferase (Wedge and Newlands (1996) *Br J Cancer* **73**: 1049) will increase that risk. Thus the alternative of increasing the effectiveness of Temozolomide by pretreatment with thioguanine may be a preferable course of action.

### P17 Mismatch repair protein expression in response to treatment with cisplatin

*A Coupe, M Burgess, A Hancock and M Mott*
Institute of Child Health, Royal Hospital for Sick Children, St Michael's Hill, Bristol BS2 8BJ, UK

Cisplatin induces DNA adducts, which are recognized by proteins of the mismatch repair system. Loss of these proteins is implicated in the development of resistance. This occurs either directly due to failure to recognize damaged DNA or indirectly by permitting mutational events in unrelated genes which in turn confer resistance. Evidence supports the former and suggests a role for the mismatch repair system in initiating apoptosis. The aim of this study was to determine if the expression of hMSH2, hMLH1, and hPMS2 protein altered in response to cisplatin exposure and to determine the relationship, if any, to that of p53 and p21. Following continuous exposure to cisplatin, protein lysates were prepared from a variety of mismatch proficient and deficient cell lines. Cells were harvested at either defined intervals over 24 h following an $IC_{75}$ dose of cisplatin or at 24 h following a dose–response. Apoptosis was determined by propidium iodide staining and flow cytometry. Protein levels were identified by Western blotting and chemiluminescent detection and subsequent densitometry. Cisplatin-induced apoptosis was demonstrated for all cell lines. Increasing p53 and p21 protein levels were found in all mismatch-deficient and proficient cell lines with respect to cisplatin dose and time of exposure reaching a maximum at 16–24 h. Conversely, the levels of MSH2, MLH1 and PMS2, where present, remained unaltered. This indicates that in contrast to p53 and p21, whole cell levels of hMSH2, hMLH1, and hPMS2 are independent of cisplatin damage.

### P18 Effect of tumour size and tumour type on passive tumour accumulation of polymeric anticancer agents

*YN Sat[1], M Bibby[2] and R Duncan[1]*
Centre for Polymer Therapeutics, School of Pharmacy, 29–39 Brunswick Square, London WC1N 1AX, UK; [2]University of Bradford, Bradford, UK

Tumour vasculature differs from normal vessels in that the endothelium is frequently discontinuous and it lacks a basement membrane (Jain, 1987). The resultant selective extravasation of circulating soluble macromolecules (polymers and proteins) and liposomes affords a means to target drugs selectively to solid tumours by a mechanism termed 'enhanced permeability and retention' (EPR) effect (Matsumara and Maeda, 1986; Duncan et al, 1996). Due to the known inherent differences in tumour vasculature of different tumour types, and also different vascularization states at different stages of tumour development, it is important to calibrate experimental tumour models according to their capacity to capture macromolecules by the EPR effect. Understanding the EPR effect is also vitally important in the context of targeting molecular medicines, e.g., gene therapy. Here, HPMA copolymer-doxorubicin (PK1) and Evans blue dye were used as probes to define the EPR effect in different tumour types (murine and human tumour xenografts) and tumours of different size at 1 h after intravenous administration.

In B16F10 murine melanoma, total PK1 accumulation was highest when the tumour size was smallest (13.7 ± 3.6 % dose $g^{-1}$, < 0.1 g). Accumulation decreased as tumour size increased (1.1 ± 1.0 % dose $g^{-1}$ > 1 g). Using Evans blue as probe, a similar accumulation pattern was observed in the small and large tumours respectively (16.5 ± 9.5 % dose $g^{-1}$, <0.1 g; 2.7 ± 0.9 % dose $g^{-1}$, >0.9 g). In MAC 15A and MAC 26 models there was no difference in tumour accumulation of PK1 with increasing tumour size and uptake was 8–11 % dose $g^{-1}$). In human tumour xenografts, PK1 displayed highest accumulation in a SK-N-SH (8.1 ± 2.9 % dose $g^{-1}$), followed by COR L23 (7.8 ± 1.1 dose % $g^{-1}$) and then SK-N-DZ (3.3–3.7 % dose $g^{-1}$). In addition to displaying different degrees of PK1 uptake, the tumours displayed different capacities to release free doxorubicin from the conjugate (doxorubicin release is mediated by lysosomal thiol proteases). Extent of polymer capture by the EPR effect, and differences in intratumoural enzyme level would be expected to effect anti-tumour activity and hence the clinical outcome.

#### REFERENCES

Duncan R, Dimitrijevic S and Evagorou E (1996) *STP Pharma* **6**: 237
Jain R (1987) *Cancer Res* **48**: 2641
Matsumura Y and Maeda H (1986) *Cancer Res* **46**: 6387

© 1999 Cancer Research Campaign

### P19 Evidence for nitrosated glycine derivatives as a source of DNA damage in man

KL Harrison[1,2] and DEG Shuker[1]
[1]MRC Toxicology Unit, University of Leicester, Leicester LE1 9HN, UK;
[2]School of Epidemiology and Health Sciences, University of Manchester, Manchester M13 9PT, UK

Raised levels of alkyl-DNA adducts have been associated with increased risk of cancers in man. In particular, $O^6$-methylguanine ($O^6$-MG) is elevated in DNA from individuals at high risk of GI tract cancers. Recently we have shown that nitrosated glycine derivatives such a N-nitrosoglycocholic acid gives rise to $O^6$-MG (Shuker and Margison, *Cancer Res* (1997) **57**: 355) as well as the expected $O^6$-carboxymethylguanine ($O^6$-CMG), (Harrison et al (1997) *Chem Res Toxicol* **10**: 652). $O^6$-CMG is a useful marker of nitrosated glycine derivatives and a sensitive immunoslot blot method has been developed for use in experimental and human studies. Administration by gavage of potassium diazoacetate (nitrosated glycine) to rats resulted in dose-dependent and persistent increases in $O^6$-CMG in stomach DNA. $O^6$-CMG was detected in untreated animals at a level of 20 $O^6$-CMG $10^{-7}$ G. Interestingly, $O^6$-CMG was also detected at similar levels in both gastric DNA (18–104 adducts $10^{-7}$ G) and peripheral lymphocyte DNA (6–250 adducts $10^{-7}$ G) from patients attending a dyspepsia clinic (in collaboration with Prof. ATR Axon and Dr S Everett, Centre for Digestive Diseases, Leeds General Infirmary, Leeds). Taken together, the detection of increased levels of $O^6$-CMG adducts in stomach DNA from experimental animals exposed to potassium diazoacetate and the detection of the same adduct in both unexposed animal and human gastric DNA suggests that nitrosated glycine derivatives may be the responsible agents.

Funding from the Medical Research Council and MAFF is gratefully acknowledged.

### P20 Highly sensitive and rapid immunoassay for the quantitation of imidazole ring opened N7-methyldeoxyguanosine in human DNA

K Harrison[1,2], K Haque[1], GP Margison[1] and AC Povey[2]
[1]Department of Genome Damage and Repair, CRC Paterson Institute, Manchester M20 9BX, UK; [2]School of Epidemiology and Health Sciences, Manchester University, Manchester M13 9PT, UK

N7-methyldeoxyguanosine (N7-medGua) could be a useful biomarker of recent past exposure to environmental and endogenous methylating agents for use in epidemiological studies (Saffhill et al (1985) *Biochem Biophys Acta* **832**: 111). In order to investigate the presence of N7-medGua in human samples obtained from routine surgical biopsies it is necessary to have a sensitive assay system that requires the minimum amount of DNA (< 10 μg). To this end polyclonal antibodies against the chemically stabilized imidazole ring-opened form of N7-medGua (RON7-medGua) have been used to develop a highly sensitive immunoslot blot assay adapted from a method by van Delft et al ((1994) *Carcinogenesis* **15**: 1867).

DNA which had been methylated in vitro with *N*-methyl-*N*-nitrosourea was used to generate a standard of known N7-methyldGua concentration as determined by $^{32}$P-post-labelling (Haque et al (1994) *Carcinogenesis* **15**: 2485). This was then diluted with various amounts of unmethylated calf thymus DNA to generate a series of standards. The immunoslot blot assay afforded a limit of quantitation of 0.097 μmol RON7-medGua/mol deoxyguanosine (dGua) using only 1 μg per analysis enabling samples to be run in duplicate or triplicate on at least one occasion. Method validation was carried out on pyloric DNA of rats treated with *N*-methyl-*N*´-nitro-*N*-nitrosoguanidine, for which N7-medGua levels had already been quantitated by $^{32}$P-post-labelling. The results showed a high degree of correlation between the two assays ($r$ = 0.99). N7-medGua was analysed in DNA samples contaminated with known amounts of RNA to investigate the possible interference of 7-methylguanosine, which is a naturally occurring minor nucleoside in RNA (Bianchini et al (1993) *Carcinogenesis* **14**: 1677). Up to 0.5 μg RNA 1 $\mu g^{-1}$ DNA did not result in an increased background signal. The immunoslot blot assay was then applied to a range of human DNA samples of individuals not known to be exposed to methylating agents. Levels of N7-medGua where detected in the range of 0.107–1.34 μmol RON7-medGua/mol deoxyguanosine in bladder tumour, colon carcinoma and colorectal mucosa DNA samples.

This rapid technique allowing for up to ten samples in triplicate to be run on each blot should enable the determination of the extent of human exposure to both environmental (diet) or endogenous sources of methylating agents in normal colorectal tissues.

### P21 Inhibition of cell division and induction of apoptosis in melanoma cells by a mycobacterial cell wall complex

MC Filion, I Ménard, HE Phillips and NC Phillips
Bioniche Inc., 383 Sovereign Road, London, Ontario, Canada N6M 1A3 and the Faculté de pharmacie, Université de Montréal, C.P. 6128, succursale Centre-ville, Montréal, Québec, Canada H3C 3J7

Mycobacterial cell wall complex (MCC) is a cell wall composition prepared from the non-pathogenic bacterium *Mycobacterium phlei* where mycobacterial DNA is complexed on the cell wall surface. We have previously shown that in addition to stimulating macrophage cytokine synthesis, MCC inhibits the division of and induces apoptosis in human bladder transitional carcinoma cells (Filion et al (1998) *Br J Cancer*, in press). In this study we have evaluated the ability of MCC to inhibit cell division and induce apoptosis in melanoma cells. Adherent murine B16 melanoma cells were treated with MCC for 24–72 h. Significant dose-dependent inhibition of cell division (determined by the reduction of MTT) was observed in the dose range 0.1–100 μg $ml^{-1}$ MCC. No direct cytotoxicity (determined by the release of the cytoplasmic enzyme lactate dehydrogenase into the culture medium) was observed following MCC treatment. Flow cytometric analysis using propidium iodide showed that inhibition of cell division was associated with an accumulation of cells at the S to G2 transition phase of the cell cycle. Interleukin-1β-converting enzyme (ICE/caspase-1), a cysteine protease involved in the sequential activation of the caspase cascade required for apoptosis, is rapidly and transiently activated by various pro-apoptotic stimuli. Incubation of B16 melanoma cells with MCC resulted in a significant dose-dependent increase in the level of ICE/caspase-1 activity (determined using the fluorogenic substrate Z-Tyr-Val-Ala-Asp[OMe]-7-amino-4-methylcoumarin) 3 h post-treatment. Analysis of B16 melanoma DNA by agarose gel electrophoresis following MCC treatment showed the presence of a 180–200 base

© 1999 Cancer Research Campaign pair DNA ladder. These results demonstrate that MCC possesses a chemotherapeutic-like activity by inhibiting the division of B16 melanoma cells in the absence of immune effector cells. The observation that inhibition of cell division is associated with ICE/caspase-1 activation and results in DNA fragmentation characteristic of apoptosis indicates that MCC-containing formulations may have application in the treatment of malignant melanoma.

### REFERENCE

Filion MC, Lépicier P, Morales A and Phillips NC (1998) *Mycobacterium phlei* cell wall complex directly induces apoptosis in human bladder cancer cells. *Br J Cancer* (in press)

© 1999 Cancer Research Campaign 

